# A yeast-based platform for etoposide production via yatein bioconversion

**DOI:** 10.1016/j.mec.2026.e00280

**Published:** 2026-05-22

**Authors:** Nicolas Gautron, Jennifer Perrin, Ana Luisa Lopez-Vazquez, Céline Melin, Marianne Unlubayir, Killian Tiger, Cyril Nicolas, Clément Cuello, Marc Clastre, Nicolas Papon, Nathalie Giglioli-Guivarc’h, Loïc Guillonneau, Isabelle Gillaizeau, Christophe Hano, Marc Jillian, Sébastien Besseau, Vincent Courdavault

**Affiliations:** a Biomolécules et Biotechnologies Végétales, UR2106, Université de Tours, 37200, Tours, France; b Université d’Orléans, CNRS, UMR 7311, ICOA, 45067, Orléans, France; c Université d’Angers, Université de Brest, IRF, SFR ICAT, 49000, Angers, France; d Axyntis - Avenue du 11 Novembre 1918, 45300, Pithiviers, France

**Keywords:** Bioproduction, Cell factories, Metabolic engineering, Bioreactor, Etoposide, Lignan

## Abstract

Etoposide, a semisynthetic derivative of podophyllotoxin originally isolated from the rhizomes of mayapple (*Podophyllum peltatum*) and indian Podophyllum (*P. hexandrum*) plants, is one of the most powerful chemotherapeutic agents used to treat various types of solid tumors and blood malignancies. Despite its clinical importance, its supply is recurrently constrained due to a heavy reliance on plant extraction, where low natural precursor abundance and increasing climate-related pressures limit production scalability. Developing alternative manufacturing routes has therefore become a major objective, though reconstruction of this complex biosynthetic pathway has long posed significant challenges, even with recent advances in synthetic biology and metabolic engineering. Yeast has emerged as a robust cellular chassis for reconstituting, either partially or entirely, plant secondary metabolite pathways, and enabling cost-effective bioproduction. Here, we established an integrated biotechnological strategy for the sustainable production of advanced etoposide intermediates using engineered yeast cell factories. By combining pathway refactoring, gene copy number optimization, and tailored co-enzyme compatibility, we established an efficient heterologous pathway converting yatein into (−)-4′-desmethyl-epipodophyllotoxin (4′dEPT) in yeast. Iterative strain engineering improved metabolic flux distribution, leading to enhanced titers and accelerated production kinetics, while process engineering proved essential to maximizing overall system performance. Finally, we also demonstrated the viability of coupling bioproduction in cell factories with downstream, semisynthetic conversion by successfully isolating bioreactor-derived 4′dEPT and converting it into etoposide. In parallel, identifying resilient plant resources that can accumulate high levels of YAT provides a complementary strategy for securing the precursor supply at scale. Overall, this report validates the concept of a hybrid etoposide production platform integrating controlled plant biomass sourcing, engineered yeast cell factories, and chemical transformation steps.

## Highlights

• Engineered yeast enable efficient bioconversion of (−)-yatein into 4′dEPT.
• Gene ratio and CPR pairing control metabolic flux and productivity.
• Bioreactor feeding strategies intensify lignan bioproduction kinetics.
• Juniperus biomass identified as a scalable renewable yatein source.
• Hybrid bio-synthetic route supports sustainable etoposide manufacturing.

## Graphical abstract

**Figure:**
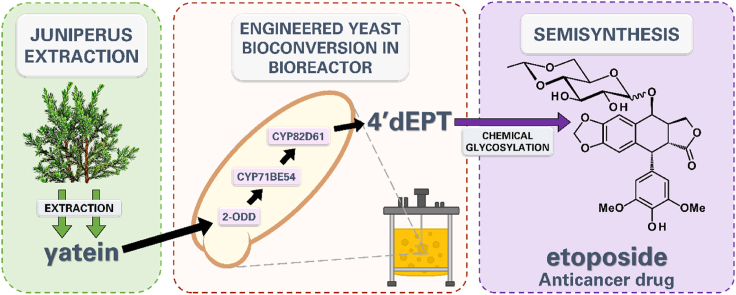

## 1 Introduction

**Fig. 1:**
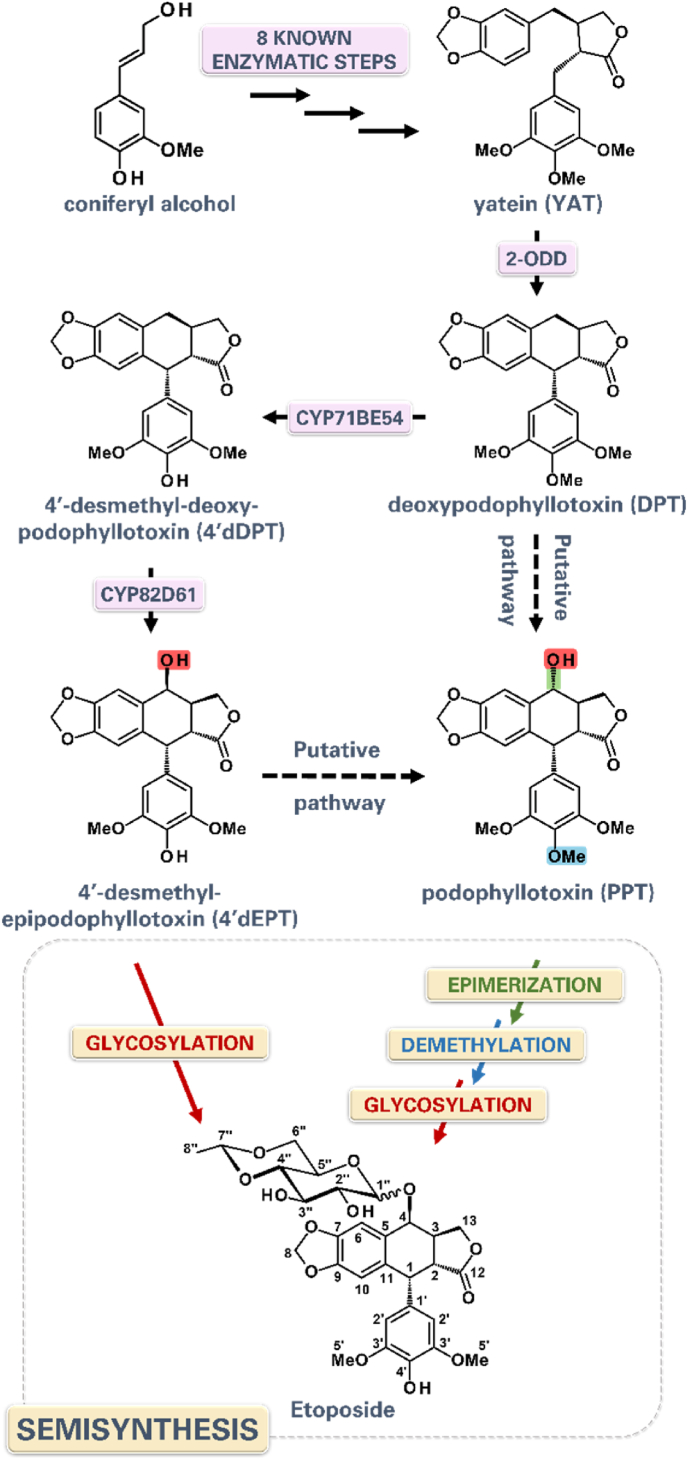
**Biosynthetic pathway from coniferyl alcohol to podophyllotoxin (PPT) and 4’desmethyl-epipodophyllotoxin (4’dEPT) with final semisynthesis for anticancer compound Etoposide.** Enzymes responsible for known steps are shown in pink. For final semisynthesis green indicates epimerization, blue indicates demethylation, red indicates glycosylation. Solid black arrows designate known pathway. Dashed black arrows designate uncharacterized putative steps for podophyllotoxin (PPT) biosynthesis from deoxypodophyllotoxin (DPT) or 4’desmethyl-epipodophyllotoxin (4’dEPT). Abbreviations stands for: 2-oxoglutarate/Fe(II)-dependent dioxygenase (2-ODD); cytochrome P450 (CYP71BE54), (CYP82D61); methyl group (Me).

Plant natural products play a pivotal and enduring role in human pharmacopoeia. These specialized metabolites indeed form an impressive array of structurally diverse and biologically potent compounds that constitute the main active molecules in many drugs. Several frontline therapeutics currently used in oncology thus originate directly or indirectly from plants. These include the anticancer agents paclitaxel (Taxol®), a taxane from the yew tree, vincristine, an alkaloid produced by the Madagascar periwinkle and etoposide, a lignan-derived molecule from the may apple (Courdavault et al., 2020). Etoposide derives from podophyllotoxin which is well known for acting as an inhibitor of topoisomerase II and is used for decades to treat lung cancers (Fig. 1) (Aisner and Lee, 1991; Jang et al., 2025). These metabolites are hallmarks of the unique capacity of plants to synthesize complex chemical scaffolds that are difficult to access through purely synthetic routes, at least on an industrial scale. Consequently, despite their significant clinical importance, the production of many of these drugs still depends heavily on extracting low-abundance metabolites from slow-growing or geographically limited plant species, as well as on semisynthetic processes that rely on plant-derived precursors. These production processes are therefore inherently limited by the low *in planta* metabolite yields, which are increasingly affected by climate change. This results in fragile supply chains that have repeatedly led to shortages globally, as observed with vincristine and etoposide, but also generates high pressure on natural plant resources thereby promoting deleterious overexploitation. For instance, the Indian Podophyllum (*Podophyllum hexandrum*) has been classified as endangered on the red list of threatened species of the International Union for Conservation of Nature (Chauhan, 2024). Importantly, the global etoposide market size is experiencing steady growth, driven by its critical role in the treatment of various cancer types. The market size was estimated at approximately USD 1.2 billion in 2024 and is projected to expand at a compound annual growth rate (CAGR) of 5–7% over the next decade, reaching around USD 2.0 billion by 2033 (“Etoposide Market Size, Share & Forecast,” n.d.). Therefore, there is an urgent need to implement sustainable and scalable alternative production strategies to secure the access to high-value plant-derived pharmaceuticals (Courdavault et al., 2021).

From this perspective, microbial cell factories tailored through synthetic and metabolic engineering strategies have emerged as a powerful alternative for overcoming the limitations of sourcing valuable plant specialized metabolites (Courdavault et al., 2020). Among microbial hosts, yeast, particularly *Saccharomyces cerevisiae,* has proven to be well-suited for metabolite bioproduction through the heterologous reconstruction of complex plant biosynthetic pathways (Guirimand et al., 2021). Building on the pioneering and successful landmark productions of hydrocortisone and the antimalarial semisynthetic artemisinin achieved in the 2000s (Szczebara et al., 2003; Paddon et al., 2013), many microbial cell factories have been next developed to produce valuable plant natural products including opioids, cannabinoids, tropane alkaloids, vinca alkaloids, saponins, and taxanes (Paddon et al., 2013; Li et al., 2018; Luo et al., 2019; Srinivasan and Smolke, 2020; Zhang et al., 2022; Liu et al., 2024; Liang et al., 2025). While many of the resulting cell factories only synthesize low amounts of the desired metabolites, the production of the analgesic thebaine by Antheia has recently reached industrial scale (Galanie et al., 2015).

Conversely, only a few examples of lignan production in cell factories have been reported to date (Kim et al., 2009; Decembrino et al., 2021; Chen et al., 2025). This limited number undoubtedly reflects the complexity of lignan biosynthetic pathways, involving enzymes with unique features, as illustrated by podophyllotoxin and its derivatives. The biosynthesis of these compounds begins with the regio- and stereoselective dimerization of two coniferyl alcohol moieties to form (+)-pinoresinol (Davin et al., 1997). (−)-yatein (YAT) derived from (+)-pinoresinol, which is converted after three reduction steps into (−)-matairesinol by two enantiospecific enzymes: (+)-pinoresinol-(−)-lariciresinol reductase (PLR) and (−)-secoisolariciresinol reductase (SDH). The intermediates between (−)-matairesinol and YAT, however, remain hypothetical, as does the order of the enzymatic reactions. Its formation nevertheless requires hydroxylation at C5, O-methylation at C4 and C5, and the formation of a methylenedioxy bridge at C3′‒C4′. In the cow parsley (*Anthriscus sylvestris*), YAT is thought to be formed from matairesinol via a biosynthetic sequence involving thujaplicatin, 5-methylthujaplicatin, and 4,5-dimethylthujaplicatin, as observed in feeding experiments using 13C- and 2H-labeled precursors (Sakakibara et al., 2003). A cDNA encoding for an *O*-methyltransferase (OMT) involved in the methylation of thujaplicatin to 5-methylthujaplicatin during yatein biosynthesis was also isolated from an *A. sylvestris* cDNA library and characterized (Ragamustari et al., 2013). Several orthologs of this OMT, as well as CYP81Q (yatein synthase), have also been identified in the flax genome (unpublished results). This metabolic pathway apparently differs from that proposed by Hendrawati et al. (2011) in *Linum* species (including *Linum usitatissimum*), as well as in the works of Marques et al. (2014) and Lau and Sattely (2015), where (−)-pluviatolide is proposed as an intermediate in podophyllotoxin biosynthesis route in *Podophyllum* species. To date, it is therefore unclear whether the biosynthesis of YAT from (−)-matairesinol follows a single, species-specific pathway resulting from convergent evolution, or whether it is organized according to a complex metabolic network.

Next, a 2-oxoglutarate-dependent dioxygenase (2-ODD) catalyzes the oxidative ring closure of YAT, leading to the formation of the aryltetralin scaffold characteristic of (−)-deoxypodophyllotoxin (DPT). Finally, two cytochromes P450, CYP71BE54 and CYP82D61, catalyze successive demethylation and hydroxylation reactions, yielding (−)-4′-desmethyl-deoxypodophyllotoxin (4′dDPT) and then (−)-4′-desmethyl-epipodophyllotoxin (4′dEPT) (Lau and Sattely, 2015) (Fig. 1). While additional enzymatic steps are required for the biosynthesis of (−)-podophyllotoxin, this compound is currently extracted from *Podophyllum* species rhizomes and converted into etoposide through a semisynthetic process, notably involving glycosylation (Canel et al., 2000) (Fig. 1).

The production of early biosynthetic intermediates of podophyllotoxin in cell factories can reach several hundred milligrams per liter (Luelf et al., 2024; Qiao et al., 2024), whereas the synthesis rates for more complex lignans remain dramatically lower. This is likely due to the high metabolic burden imposed by long biosynthetic pathways and enzyme promiscuity (Chen et al., 2025). In this context, producing podophyllotoxin or related compounds through the bioconversion of a highly bioavailable precursor stands out as an appealing alternative strategy, as recently illustrated with the synthesis of a vincristine precursor (Kulagina et al., 2021). In this study, we developed an integrated etoposide production pipeline starting with YAT as a means to propose a sustainable alternative to *Podophyllum* exploitation. This approach relies on identifying a cultivated plant that accumulates large amounts of the precursor YAT, engineering yeast cell factories that efficiently convert YAT into 4′dEPT, optimizing fermentation conditions, and finally, glycosylating 4′dEPT to yield etoposide.

## 2 Materials and methods

### 2.1 Plasmid construction

The coding sequence of CPR2 (PZ369892), 2ODD (KT390173), CYP71B54 (KT390179) and CYP82D61 (KT390182) from *P. hexandrum*, together with CPR2 from *Catharanthus roseus* (X69791) and *A. thaliana* (NM_001342001.1), were amplified by PCR from cDNA using Phusion High-Fidelity DNA polymerase (Fermentas) and gene-specific primers (Supplemental Table S1) were used to introduce *Spe*I, *Nhe*I, or *Xba*I compatible restriction sites. Codon-optimized sequences of PhCYP82D61 (Supplemental Fig S7) designed using COOL (Chin et al., 2014) and GENEius software (Eurofins) were synthesized by Eurofins and then PCR amplified using the same conditions. The resulting PCR fragments were subsequently cloned into the template vectors pDPTA125, pDPTA111, pDPAC124, pPETA104, pPETA102, pPEAC115, pDPAC112 and pDPAC113 using *Spe*I or *Nhe*I restriction sites, under constitutive promoters as specified in Supplemental Table S2 (Kulagina et al., 2021). Bacterial clones were selected on LB-kanamycin plates (50 μg mL^−1^, Duchefa Biochemie) and screened for insertion using colony PCR.

### 2.2 Yeast strains

Yeast strains were constructed using *Saccharomyces cerevisiae* CEN.PK113-7D (MATa MAL2-8C, SUC2) as parental strain. Expression cassettes were integrated into yeast hot-spot loci (Mikkelsen et al., 2012) using a CRISPR–Cas9-based method (Kulagina et al., 2021). Yeast strains harboring the pCfB2312 vector were co-transformed with gRNA helper plasmid and the appropriate NotI-linearized template vector, using the lithium acetate transformation method (Chen et al., 1992). Yeast transformants were selected on YPD medium plates (20 g.L^−1^ of peptone from meat, enzymatic digest, Sigma-Aldrich; 10 g.L^−1^ of yeast extract, Thermo Scientific Chemicals; and 20 g.L^−1^ of glucose, Euromedex France) supplemented with G418 (200 μg mL^−1^, Gibco) and nourseothricin (100 μg mL^−1^, Jena Bioscience), and screened for effective integration of each expression cassette by colony PCR. Yeast strains constructed and tested in this study are listed in Supplemental Table S3.

### 2.3 Small-scale culture conditions

Yeast were grown overnight at 30 °C in 5 mL of YPD (10 g.L^−1^ yeast extract; 20 g.L^−1^ peptone; 20 g.L^−1^ glucose). The resulting cultures were used to inoculate 200 μL of fresh YPD to an initial OD_600_ of 1, and YAT was added at final concentrations ranging from 40 to 210 mg.L^−1^. Cultures were incubated at 30 °C under shaking at 150 rpm. To maintain carbon and nutrient availability, cultures were supplemented with 20 μL of glucose 20% after 24 h and 48 h of growth, and with 10 μL of YP 10X after 48 h. Samples were collected and centrifuged at 14,000 × g for 5 min to remove cells. The clarified supernatants were diluted 1:15 in methanol (Fischer Chemical) prior to lignan analysis by UPLC–MS/DAD.

### 2.4 Baffled erlenmeyer flask culture conditions

The yeast strains were first cultured overnight in 5 mL YPD and used to inoculate 30 mL of fresh YPD at 10% (v/v) in 250 mL baffled flasks. After an initial 15 h cell growth, the cultures were supplemented with 20 mg.L^−1^ of YAT, glucose up to 20 g.L^−1^ and further incubated at 30 °C under constant agitation at 120 rpm. Samples were collected throughout the experiment. Aliquots were used directly for biomass estimation and cell viability assessment. The remaining volume of aliquots were centrifuged at 4000 × g for 4 min. The resulting supernatant was analyzed either undiluted for fermentation metabolites quantification or after centrifugation at 10,000 × g for 10 min and dilution (1:2 to 1:10) in 90% acetonitrile (Fischer Chemical) containing 0.1% formic acid (Honeywell Fluka) for lignan analysis by UPLC-MS/DAD.

### 2.5 Bioreactor culture conditions

Three steps of propagation were carried out in YPD from a single colony to inoculate the fed-batch cultures. Precultures were grown in 5 mL media in 15 mL Greiner culture tubes, 30 mL in 200 mL baffled Erlenmeyer flasks, and 150 mL in 1 L baffled Erlenmeyer flasks, with agitation adjusted to 180, 120, and 110 rpm, respectively. All cultures were incubated at 30 °C for 14 h, allowing consistent physiological adaptation across scales. Each step was used at a 10% (^v^/_v_) ratio to inoculate each next-step.

Fed-batch cultures were performed in 2 L bioreactors (Global Process Control) with an initial volume of 1.25 L, managed with the CBio2 software. The temperature was regulated at 30 °C and pH at 6.0 by the addition of 5 M KOH solution (Fischer Chemical). Air flow and stirring rate were adjusted to maintain fully aerobic conditions, i.e. a dissolved oxygen concentration above 20% of saturation. The initial medium composition contained 10 g.L^−1^ yeast extract and 20 g.L^−1^ peptone. Feeding with nutritive elements was ensured by pumping solutions of glucose (400 g.L^−1^) for growth or concentrated YPD (50 g.L^−1^ yeast extract; 100 g.L^−1^ peptone; 200 g.L^−1^ glucose) for bioconversion. Feeding with YAT was ensured by either pulsing or pumping a 20 g.L^−1^ solution, both prepared in pure ethanol (Fischer Chemical). Two strategies of bioproduction were established, combining pulsed or continuous feeding with YAT. For scale-up studies, pulsed and continuous feeding were tested with total YAT supplies of 3 × 20 mg.L^−1^ and 60 mg.L^−1^, respectively. For both process optimization studies, a continuous feeding strategy was applied with total YAT supplies of 2 g. Samples were collected throughout the experiment and processed as described above for flask cultures.

### 2.6 Fermentation metabolite analysis

Determination of glucose, ethanol, glycerol and organic acids from supernatant was performed by HPLC (Waters 600 controller, Waters 717plus autosampler), using an Aminex HPX-87H + column (300 mm ∗ 7.8 mm) set at 50 °C, a 5 mM H_2_SO_4_ as eluent at a flow rate of 0.5 mL min^−1^ and dual detection by refractometer (Waters 2414 RI detector) and UV at 210 nm (Waters 996 PDA detector).

### 2.7 Biomass quantification

Yeast biomass was estimated by performing spectrophotometric measurements (JENWAY 6320D Spectrophotometer) at 620 nm after a calibration against cell dry weight determination to evaluate yeast growth. For cell dry weight determination in bioreactors, culture medium was harvested and filtered on 0.45 μm pore-size polyamide membranes (Sartorius), which were then dried to a constant weight at 60 °C under partial vacuum.

### 2.8 Cell viability assessment

Cell coloration with methylene blue method was used to determine the cell viability (Nielsen et al., 1991). 50 μL of 0.3 mM methylene blue (Sigma Aldrich) was mixed with 50 μL of a diluted raw sample and incubated at room temperature for 10 min. A minimum of 200 cells was counted in at least five different squares of a double-side Malassez counting chamber and 150 cells in at least eight different squares of a double-side Thomas counting chamber. Cell viability was defined as the number of unstained cells (i.e. living cells) divided by the total number of cells (stained and unstained). Buds have been taken into account when they seized more than half of the parent-cell.

### 2.9 Lignan extraction from plant tissues

Samples were collected, flash-frozen in liquid nitrogen, and either directly used for metabolite extraction or freeze-dried for extraction from dry material. For metabolite extraction, samples were ground into a fine powder and sonicated in methanol. After centrifugation, the clarified extracts were diluted (1:5 to 1:20) in methanol, and 5 μL were injected into the UPLC-MS/DAD system.

### 2.10 Lignan analysis

Lignans produced from all culture systems were analyzed using an ACQUITY UPLC system (Waters) equipped with a diode array detector (DAD) and coupled to an SQD2 mass spectrometer (Waters) operating with an electrospray ionization (ESI) source. The system was controlled using MassLynx v4.2 software (Waters). Chromatographic separation was performed on an Acquity HSS T3 C18 column (150 × 2.1 mm, 1.8 μm; Waters) at 55 °C with a flow rate of 0.4 mL min^−1^. The mobile phase consisted of water (solvent A) and acetonitrile (solvent B), both containing 0.1 % formic acid. Compounds were separated using an 18 min linear gradient from 80 % to 25 % solvent A. Mass spectrometric detection was performed in positive ionization mode. The capillary voltage was set to 3.0 kV and the cone voltage at 30 V. Cone and desolvation gas flow rates were set to 60 and 800 L h^−1^, respectively. Lignans were quantified from UV chromatograms using calibration curves generated from commercial YAT (Ambinter) and 4′dEPT (Biosynth) standards. DPT and 4′dDPT were quantified using the YAT calibration curve and expressed as YAT equivalents.

### 2.11 YAT and 4’dEPT purification

To obtain a large amount of YAT, a total of 12.8 kg of *Juniperus* spp. fresh aerial parts (*J. sabina, J. procumbens, J. scopulorum, J. × media, J. pfitzer* and *J. horizontalis* purchased from Promesses de fleur (France, https://www.promessedefleurs.com/)) were mechanically ground and extracted with 3 L of methanol per kg biomass for 4 h at 60 °C under sonication. After Büchner filtration, the plant residue was re-extracted overnight under identical conditions. Combined extracts were concentrated to dryness under reduced pressure at 40 °C, affording 1.4 kg of crude extract. To conduct a silica pre-purification, the extract was dissolved in 6 L of methanol and impregnated onto 3 kg of silica gel (Sigma Aldrich). The dry and impregnated silica was loaded onto lina and percolated with ethyl acetate (42 L, Fischer Chemical). Concentration under reduced pressure afforded 490.9 g of pre-purified extract. The extract was dissolved in 1.5 L ethyl acetate and purified by normal-phase chromatography on a DAC LC200 column packed with 5 kg Chromatorex GS60 (20–45 μm) in ethyl acetate at 45 bars, eluted with heptane/EtOAc (80:20, v/v, Fischer Chemical). Fractions were collected and analyzed for YAT content (UPLC-DAD/MS). Combined YAT-containing fractions yielded 77 g after solvent removal. Further purification was performed by reverse-phase chromatography on a DAC LC200 column packed with 5 kg Chromatorex C18 (SPS100-10HE) in acetonitrile, eluted with H_2_O/acetonitrile (60:40, v/v) containing 0.1% formic acid. Fifteen injections of 5 g each were performed and YAT was monitored at 286 nm to collect fractions. YAT-containing fractions were combined and concentrated in a REP163 reactor (160 L capacity) to a residual volume of 5 L aqueous phase. The reactor was rinsed with 40 L methanol. Aqueous and methanolic phases were separately concentrated under reduced pressure, yielding 0.7 g and 17.8 g respectively, corresponding to 18.5 g total. The enriched fraction was dissolved in 50 mL ethyl acetate, impregnated onto 50 g silica gel, and dry under reduced pressure. A final purification step was conducted by normal-phase chromatography on a DAC LC50 column packed with 425 g Chromatorex GS60 20-45 μm) in ethyl acetate. The impregnated dry-silica material was deposited directly onto the top of the column and eluted with toluene/ethyl acetate 9:1 (v/v, Fischer Chemical) followed by 6:4 (v/v). YAT was monitored at 286 nm. Combined fractions of interest were concentrated under reduced pressure to afford 12.5 g of YAT as a pale green amorphous solid.

4’dEPT was purified from two 1.4 L fermentation reactors, yielding an estimated 920 mg of the target compound. The reactor broths were extracted twice with dichloromethane (v/v, Fischer Chemical). The combined organic phases were concentrated under reduced pressure using a rotary evaporator, yielding 3.45 g of crude extract. The extract was further purified on a DAC LC50 column packed with 303 g Kromasil C18 silica (5 μm) in acetonitrile at 50 bar, eluted with H_2_O/acetonitrile (80:20, v/v) containing 0.1% formic acid. Elution was monitored at 280 nm and fractions containing 4′dEPT were combined. Drying under reduced pressure afforded 885 mg of 4’dEPT.

(5*R*,5a*R*,8a*R*,9*S*)-9-hydroxy-5-(4-hydroxy-3,5-dimethoxyphenyl)-5,8,8a,9-tetrahydrofuro[3′,4':6,7]naphtho[2,3-d][1,3]dioxol-6(5a*H*)-one (4’dEPT), isolated as a white solid, M.p.: 246‒248 °C. ^1^H NMR (400 MHz, CDCl_3_) δ = 6.87 (s, 1H, H6), 6.55 (s, 1H, H10), 6.29 (s, 2H, H2′), 5.99 (d, *J* = 11.1 Hz, 2H, H8), 5.40 (s, 1H, C4′-OH), 4.93 – 4.78 (m, 1H, H4), 4.61 (d, *J* = 5.1 Hz, 1H, H1), 4.40 – 4.32 (m, 2H, H13), 3.77 (d, *J* = 1.2 Hz, 6H, H5′), 3.26 (dd, J = 14.0, 5.1 Hz, 1H, H2), 2.90 – 2.77 (m, 1H, H3), 1.75 (s, 1H, C4-OH) ppm (Supplemental Fig S1). ^13^C NMR (101 MHz, CDCl3): δ = 175.2 (C, C12), 148.8 (C, C7), 147.7 (C, C9), 146.6 (C, C3′), 134.3 (C, C4′), 132.4 (C, C11), 132.0 (C, C5), 130.7 (C, C1′), 110.7 (CH, C10), 109.1 (CH, C6), 108.1 (2 × CH, C2′), 101.7 (CH_2_, C8), 67.7 (CH_2_, C13), 67.0 (CH, C4), 56.6 (2 × CH3, C5′), 43.9 (CH, C1), 40.8 (CH, C2), 38.4 (CH, C3) ppm (Supplemental Fig. S2). IR (neat): ṽ = 3404, 2915, 1770, 1613, 1516, 1504, 1483, 1454, 1378, 1330, 1228, 1109, 1080, 1034, 996, 928, 889, 798, 732, 698 cm^−1^.

### 2.12 Chemical synthesis of etoposide

Unless otherwise stated, all reagents and starting materials were purchased from commercial suppliers and used as received. Air and/or moisture sensitive reaction was performed under an argon atmosphere using anhydrous solvents. MeCN (99.9% GC HPLC) was purified by passage through a column packed with activated alumina under nitrogen pressure (Dry Solvent Station GT S100, Glass Technology). In a round bottom balloon under argon atmosphere, 4′-demethyl-4-epipodophyllotoxin produced in yeast (400 mg, 1 mmol) and commercially available 4,6-*O*-ethylidene-2,3-*O*-dibenzyl-D-glucose (1.2 equiv., 464 mg, 1.2 mmol) were dissolved in dry acetonitrile (9.6 mL). The solution was cooled at −10 °C. Boron trifluoride diethyl etherate (1.5 equiv., 192 μL, 1.5 mmol, Sigma Aldrich) was added, and the mixture was stirred at −10 °C for 18 h. The reaction was quenched with pyridine (360 μL, 4.5 mmol, Sigma Aldrich). The mixture was concentrated under reduced pressure. The residue was diluted in dichloromethane and washed 3 times with brine. The resulting organic phase was dried over MgSO_4_, filtered and concentrated under reduced pressure. The crude product was purified using reverse phase chromatography, RP-18 silica (25‒40 μm) H_2_O/ACN from 9:1 to 0:10 (v/v), to obtain the product as a white solid (190.5 mg, 25%). The product was obtained as a mixture of anomers β/α in a 6:4 ratio.

(5*R*,5a*R*,8a*R*,9*S*)-9-(((2*R*,4a*R*,7*R*,8*S*,8a*R*)-7,8-bis(benzyloxy)-2-methylhexahydropyrano[3,2-d][1,3]dioxin-6-yl)oxy)-5-(4-hydroxy-3,5-dimethoxyphenyl)-5,8,8a,9-tetrahydrofuro[3′,4':6,7]naphtho[2,3-d][1,3]dioxol-6(5aH)-one. ^1^H NMR (400 MHz, CDCl_3_) (anomeric mixture β/α in a 6:4 ratio) δ = 7.40 – 7.17 (p.o, 10H), 7.01 (s, 0.6H), 7.00 (s, 0.4H), 6.87 (s, 0.6H), 6.83 (s, 0.4H), 6.56 (s, 0.6H), 6.51 (s, 0.4H), 6.26 (s, 0.8H), 6.25 (s, 1.2H), 5.96 (d, *J* = 3.6 Hz, 1.2H), 5.94 (d, *J* = 30.8 Hz, 0.8H), 4.92 – 4.83 (p.o, 2.4H), 4.79 – 4.71 (p.o, 2.6H), 4.69 – 4.65 (p.o, 0.6H), 4.64 – 4.55 (p.o, 2.2H), 4.52 (d, *J* = 10.7 Hz, 0.8H), 4.42 – 4.34 (p.o, 0.8H), 4.25 – 4.14 (p.o, 1.2H), 4.12 – 4.05 (p.o, 0.4H), 3.98 (d, *J* = 5.4 Hz, 0.4H), 3.91 – 3.82 (p.o, 0.4H), 3.77 ‒ 3.73 (p.o, 6H), 3.68 – 3.62 (p.o, 0.8H), 3.59 – 3.50 (p.o, 1.2H), 3.49 – 3.42 (p.o, 1.2H), 3.42 – 3.34 (p.o, 1.6H), 3.34 – 3.19 (p.o, 1.4H), 2.93 – 2.75 (p.o, 1.2H), 1.38 (d, *J* = 4.9 Hz, 1.8H), 1.33 (d, *J* = 5.0 Hz, 1.2H) ppm (Supplemental Figs. S3 and S4). HRMS (ESI): *m/z* calcd. for C_43_H_43_O_13_ [M - H]^‒^ 767.2709, found 767.2691.

### 2.13 Structural characterization

Anomeric ratio was determined by 1H NMR analysis of the mixtures of the isolated product. NMR spectra were recorded at 298 K on a 400 MHz (Bruker Advance III HD) Nanobay spectrometer equipped with a BBO probe. Chemical shifts (δ) for ^1^H NMR (400 MHz), ^13^C NMR (101 MHz) are reported in parts per million (ppm) with coupling constants (*J*) given in Hertz (Hz). Tetramethylsilane (TMS, Sigma Aldrich) served as an internal standard, or alternatively, all 1H and ^13^C shifts were referenced to the residual solvent peak of CDCl_3_ (^1^H referenced to 7.26 ppm and ^13^C referenced to 77.16 ppm). The ^13^C was acquired in a broad-band decoupled mode. Multiplicities are designated as singlets (s), broad singlets (brs), doublets (d), triplets (t), combinations thereof (e.g., dt for a doublet of triplets), quartets (q), multiplets (m), overlapped (o) or partially overlapped (p.o). Primary NMR data files were processed using MestReNova 1D [^1^H NMR, ^13^C NMR, Distortionless Enhancement by Polarization Transfer (DEPT)] and 2D NMR experiments [Correlation Spectroscopy (^1^H−^1^H COSY and ^1^H−^13^C Heteronuclear Single Quantum Coherence (HSQC)] were employed to facilitate signal assignment. In case of ambiguous proton and carbon assignments, Heteronuclear Multiple-bond Correlation (HMBC) spectroscopy was utilized. High-resolution mass spectra were recorded using a Brucker maXis ESI qTOF ultrahigh-resolution mass spectrometer coupled to a Dionex Ultimate 3000 RSLC system (FR2708, Orléans). MS data were acquired in positive ion mode and processed with Data Analysis 4.4 software (Bruker). Data are presented in the following format: molecular formula for the calcd. fragment, calcd. fragment *m/z*, and detected fragment *m/z* (with relative ratio expressed in percent unless otherwise stated). Infrared spectra were recorded with a Thermo Scientific Nicolet IS10 FTIR spectrometer using diamond ATR golden gate sampling and are reported in wave numbers (cm^−1^). Melting points (m.p., °C) were determined using a Thermo Scientific 9200, N° IA9200X6 apparatus in open capillaries and are uncorrected. Thin-layer chromatography (TLC) was performed on precoated Merck Silica Gel 60 F254 plates, with spot visualization achieved via UV light (254 nm) and/or staining by immersion in a 1% aqueous potassium permanganate solution, followed by charring at 150 °C. Flash chromatography was conducted on RP-18 silica (25–40 μm) using technical-grade solvents with H2O, acetonitrile (ACN), or combinations thereof as eluents. The IUPAC nomenclature for new compounds was automatically generated using the structure-to-name generator included in Chem Draw 22.2.

Single crystals (Supplemental Fig. S5, suitable for X–ray crystallographic analysis, were obtained by slow evaporation of CH_2_Cl_2_-Toluene solution. X–ray diffraction experiments for monocrystal of 4’dEPT were performed at 172 K with graphite–monochromatized Mo K radiation (=0.71073 Å) on a Bruker D8 QUEST diffractometer. Formula C_21_H_20_O_8_, formula weight 400.37. Crystal system triclinic, space group P-1, a = 8.4796(6) Å, b = 11.4095(10) Å, c = 19.1076(18) Å, α = β = γ = 90°, V = 1848.6(3) Å3, Z = 4, calculated density = 1.439 g/cm^3^, = 0.111 mm^−1^, R_int_ = 0.0398, R[F22(F^2^)] = 0.0377, wR(F^2^) = 0.0936. Indepedent reflections = 5475. GOF = 1.033, 342 parameters, final difference map within 0.235 and - 0.210 eÅ^−3^. The structure was solved using direct methods and refined by full-matrix least-squares analysis on F2. Program(s) used to solve structure: SHELXT2014/5 (Sheldrick, 2015). Program(s) used to refine structure: SHELXL2019/1 (Lübben and Sheldrick, 2019). Software used to prepare material for publication: SHELXTL. CCDC 2536572 contains the supplementary crystallographic data for this paper. These data can be obtained free of charge from The Cambridge Crystallographic Data Center via www.ccdc.cam.ac.uk/data_request/cif.

## 3 Results and discussion

### 3.1 Bioproduction of 4’dEPT in engineered yeast require specific CPR

**Fig. 2:**
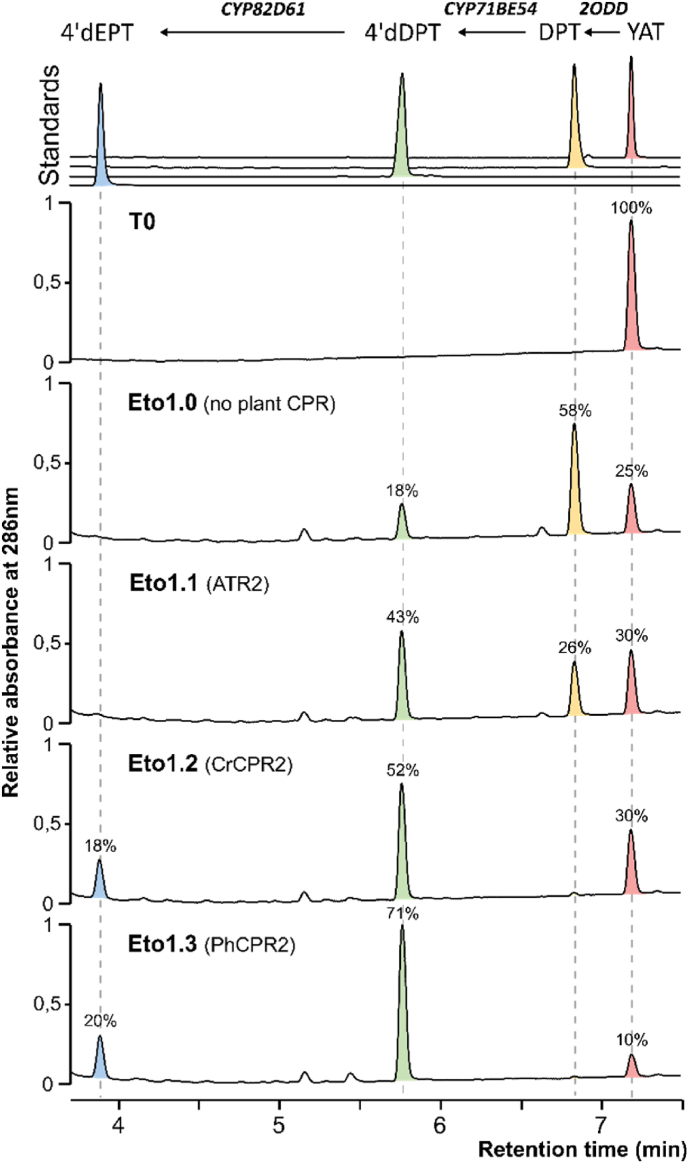
**Reconstruction in *S. cerevisiae* of the 4’dEPT biosynthesis pathway from YAT and evaluation of plant CPRs on bioconversion efficiency.** The stable yeast strain harboring single copies of 2ODD, CYP71BE54 and CYP82D61 from *P. hexandrum* (Eto1.0) was further engineered to express distinct plant CPR in addition to the yeast endogenous CPR. ATR2, CrCPR2 or PhCPR2 were stably integrated in Eto1.0 to generate strain Eto1.1, strain Eto 1.2 and strain Eto 1.3, respectively. For each strain, bioconversions were performed in 200 μL small-scale assays in YPD for 72 h after addition of 40 mg.L^−1^ YAT in the culture medium. YAT transformation was monitored in culture supernatants by UPLC-MS/DAD. UV chromatograms are presented at 286 nm with relative signal intensity and MS acquisitions at the retention time of interest are shown in Supplemental Fig. S6. The initial condition (T0) was used as control. The percentage value above each peak indicates the proportion of each compound produced in the different yeast strains.

In a first attempt to produce 4’dEPT in yeast using YAT as a precursor, a single gene copy of 2ODD, CYP71BE54, and CYP82D61 from *P. hexandrum*, each controlled by strong and constitutive promoters (Supplemental Table S3), was integrated into well-established genomic loci of *S. cerevisiae CEN.PK113-7A* (Supplemental Table S2, Mikkelsen et al., 2012), generating the Eto 1.0 strain. Yeast were grown for 72 h with 40 mg.L^−1^ of YAT supplemented in the culture medium. Bioconversion was monitored by analysing culture supernatants using UPLC-MS-DAD. As shown in the UV chromatograms (Fig. 2), the amount of YAT in the medium of Eto 1.0 decreased after 72 h compared with the initial condition (T0), together with the appearance of two new compounds eluting at 5.75 and 6.83 min retention times, respectively. The main product detected at 6.83 min, displays a mass to charge ratio (*m/z*) of 399.2 and shows a characteristic fragmentation yielding an ion at *m/z* 231.0 originating from the core structure (Supplemental Fig. S6). Such a fragmentation behavior, commonly associated with the loss of the benzene ring of arylnaphthalene lactone lignans (Li et al., 2012), is consistent with that was observed for YAT and 4’dEPT standards, and supports the annotation of this compound as DPT, the expected product of 2ODD. The second compound, eluting at 5.75 min and exhibiting an *m/z* of 385.2 with a similar fragmentation pattern was assigned to 4’dDPT, the reported product of CYP71BE54. In contrast, 4’dEPT was not detected at the retention time corresponding to the authentic standard. Altogether, these results indicate that the first enzymatic step catalyzed by 2ODD proceeds efficiently in yeast, whereas the subsequent cytochrome P450-dependent reactions are limiting or inactive.

Plant P450s expressed in yeast generally require the addition of a plant cytochrome P450 reductase (CPR) to achieve efficient electron transfer, since the endogenous yeast CPR is poorly efficient at properly reducing them (Jennewein et al., 2005). Therefore, the well-characterized and widely used *Arabidopsis* class II CPR (ATR2) was introduced into the Eto 1.0 backbone, generating the Eto 1.1 strain. The specific selection of ATR2 was guided by tight association of class II CPR with specialized metabolite biosynthesis *in planta* (Parage et al., 2016). Indeed, ATR2 clearly improved CYP71BE54 activity, resulting in the accumulation of 4’dDPT as the major product. However, 4’dEPT remained undetectable, suggesting once again a default in CYP82D61 activity under these conditions. Recent studies have indeed highlighted the strong dependence of plant P450 catalytic performance in yeast on the nature of the associated CPR and the importance of native CPR–P450 pairing for optimal activity (Liu et al., 2025). Therefore, CPR2 homologues from *Catharanthus roseus* or *P. hexandrum* were integrated in place of ATR2 in Eto 1.1, yielding the Eto 1.2 and Eto 1.3 strains, respectively. In both cases, these alternative CPR2s led to the appearance of a specific compound co-eluting with the 4’dEPT standard at 3.86 min (Fig. 2) and displaying a *m/z* of 401.2, consistent with the expected product. Notably, DPT was no longer detected in these strains whereas 4’dDPT accumulations were improved. This indicates an overall enhancement in CYP71BE54 catalytic efficiency, particularly in the presence of PhCPR2 (Eto 1.3). Thus, with the use of an adapted CPR, a critical hurdle to 4’dEPT production in yeast has been overcome, although the final CYP82D61-catalyzed step still appears to limit the overall biosynthetic flux.

### 3.2 Improving the CYP82D61 rate-limiting step in 4’dEPT biosynthesis

To improve the bioconversion efficiency of YAT into 4′dEPT, the strain Eto1.3 was selected for further engineering, with a particular emphasis in the rate-limiting step catalyzed by CYP82D61. Two codon-optimized variants of CYP82D61, adapted to yeast codon usage (Supplemental Fig. S7), were designed and introduced into the Eto 1.3 background in place of the CYP82D61 native sequence, generating the Eto 2.1 and Eto 2.2 strains.

**Fig. 3:**
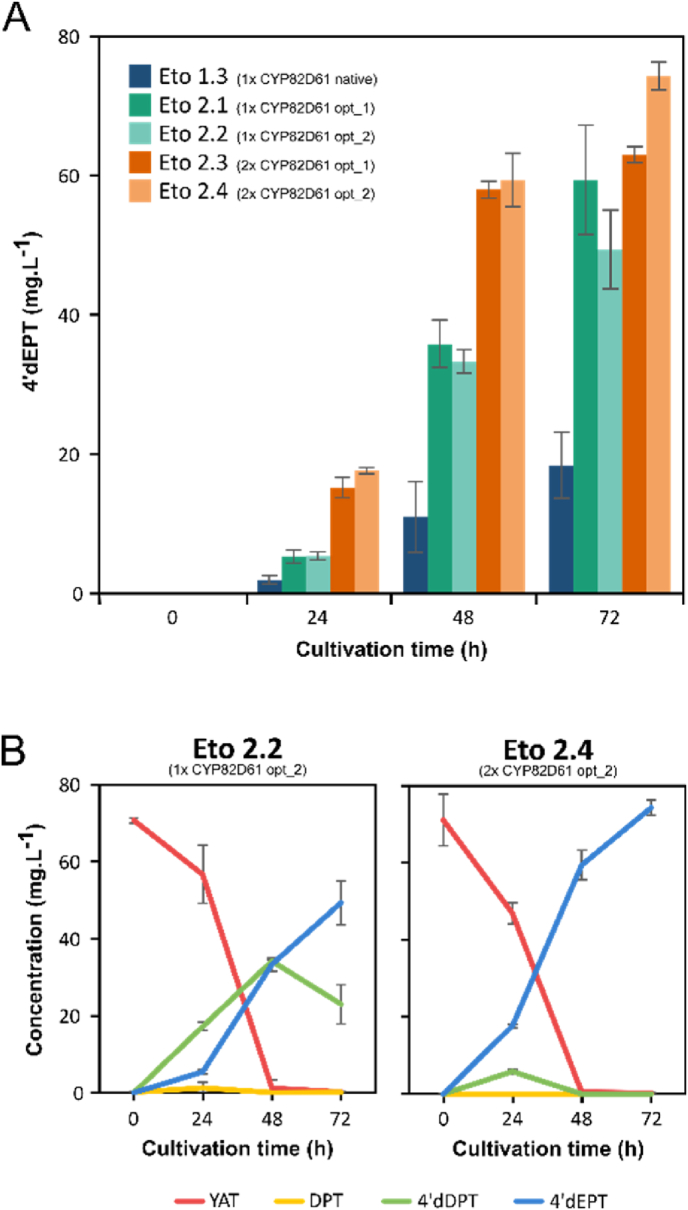
**Increased CYP82D61 expression enhances 4′dEPT production in engineered yeast.** Yeast strain Eto 1.3 expressing native sequences of 2ODD, CYP71BE54, and CYP82D61 together with PhCPR2 was compared to strains differing only in CYP82D61, in which the native sequence was replaced by codon-optimized variants (Opt1 and Opt2; Supplemental Fig. S7) expressed either as single gene copies (Eto 2.1 and Eto 2.2) or as double gene copies (Eto 2.3 and Eto 2.4). (A) Time-course production of 4′dEPT among strains. (B) Detailed time-course profiling of lignans in Eto 2.2 and Eto 2.4 strains (data for Eto 1.3, Eto 2.1, and Eto 2.3 are shown in Supplemental Fig. S8). Bioconversions were performed in 200 μL small-scale assays in YPD for 72 h after addition of 70 mg.L^−1^ YAT in the culture medium. YAT transformation was monitored in culture supernatants by UPLC-MS/DAD. Error bars represent standard deviation (n = 3 or 4 biological replicates).

Under these conditions, and starting from 71.1 ± 6.6 mg.L^−1^ of YAT supplemented in the culture medium, the reference strain Eto 1.3 produced 18.4 ± 4.8 mg.L^−1^ of 4’dEPT after 72h (Fig. 3A). In comparison, both Eto 2.1 and Eto 2.2 strains showed improved 4’dEPT bioproduction, with a 3.2 and 2.7-fold increase in titer, reaching 59.4 ± 7.8 and 49.4 ± 5.6 mg.L^−1^, respectively. However, a substantial accumulation of 4′dDPT, the substrate of CYP82D61, was still observed (Fig. 3B, Supplemental Fig. S8), indicating that this step remained a metabolic bottleneck. A second strategy was therefore implemented by increasing the genomic copy number of the optimized CYP82D61 sequences, generating the Eto 2.3 and Eto 2.4 strains. While their final 4’dEPT titers at 72 h were only slightly higher than those obtained with single-copy strains (63.0 ± 1.2 et 74.3 ± 2.0 mg.L^−1^, respectively), these strains exhibited a complete consumption of YAT and metabolic intermediates within the same time frame, consistent with a near-quantitative bioconversion of YAT **(**Fig. 3A–B, Supplemental Fig S8). Moreover, Eto 2.3 and 2.4 exhibited markedly accelerated production kinetics, with 3.3 and 1.7-fold higher conversion rates at 24 h and 48 h respectively. Altogether, these results indicate that combining codon optimization with an increased gene ratio favoring CYP82D61 enables faster pathway turnover and promotes efficient metabolic flux toward 4′dEPT biosynthesis.

### 3.3 Enhancing yeast bioconversion capacity through genes copy number adjustment

To further enhance 4′dEPT production, the concentration of precursor supplied to the culture medium was increased threefold to reach 210 mg.L^−1^ of YAT. To enable engineered yeast to cope with this elevated substrate load and to prevent the emergence of a downstream bottleneck, the genomic copy number of *CYP82D61* was increased from two to six to generate the Eto 3.1 strain. In addition, to minimize potential transcriptional silencing associated with the high gene copy number of CYP82D61, both native and codon-optimized sequences of CYP82D61 were combined to diversify transcript sequences.

Under these conditions, with 210 ± 9.1 mg.L^−1^ of YAT, the Eto 3.1 strain produced 103.9 ± 9.1 mg.L^−1^ of 4’dEPT within 72 h (Supplemental Fig. S9). However, only 42% of the supplied YAT was consumed, and no accumulation of downstream biosynthetic intermediates was detected, suggesting that pathway flux toward 4′dEPT remained efficient while substrate entry into the pathway became limiting. Therefore, to improve YAT utilization, the genomic copy numbers of the enzymes catalyzing the first two steps of the pathway (2ODD and CYP71BE54) were progressively increased, from a single copy in Eto 3.1 up to four copies in the Eto 3.4 strain.

**Fig. 4:**
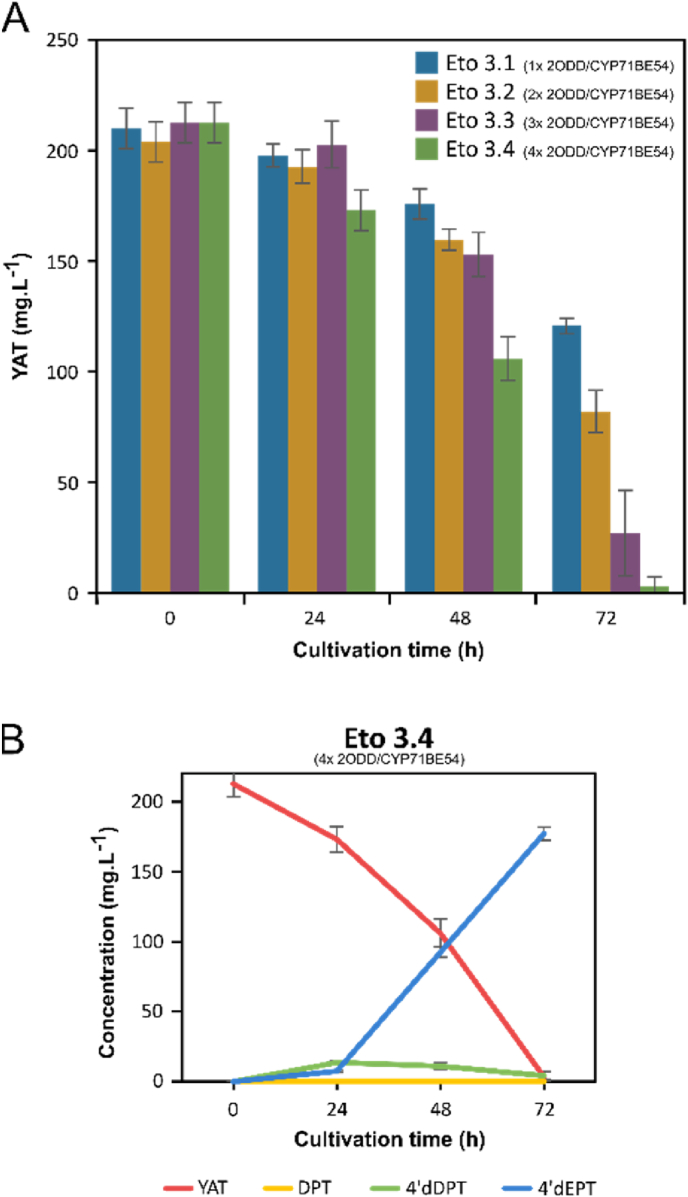
**Enhancing YAT consumption increases 4′dEPT production in engineered yeast.** The control strain Eto 3.1 harbors a single copy of 2ODD and CYP71BE54, six copies of CYP82D61 (two natives and two sets of codon-optimized variants), and PhCPR2. The copy numbers of both 2ODD and CYP71BE54 were increased stepwise, with one copy each in strain Eto 3.1, two copies each in Eto 3.2, three copies each in Eto 3.3, and four copies each in Eto 3.4 (A) Time-course consumption of YAT across strains. (B) Detailed time-course profiling of lignans in Eto 3.4 strain (data for Eto 3.1, 3.2 and 3.3 are shown in Supplemental Fig. S9). Bioconversions were performed during 72 h in 200 μL small-scale assays in YPD supplemented with 210 mg.L^−1^ YAT. YAT bioconversion was monitored in culture supernatants by UPLC-MS/DAD. Error bars represent standard deviation (n = 3 or 4 biological replicates).

Comparison of YAT consumption profiles across these strains highlighted a clear correlation with gene copy number, culminating in a complete YAT depletion within 72 h in the Eto 3.4 (Fig. 4A). Consistently, only minor accumulation of pathway intermediates was observed in this strain (Fig. 4B). This indicates that early step enhancement did not generate any new bottleneck at the CYP82D61 step, especially when six copies of the corresponding gene were present. Among all tested strains, Eto 3.4 proved to be the most efficient and displayed the fastest kinetics of 4′dEPT production, reaching 177.1 ± 4.8 mg.L^−1^ after 72 h.

### 3.4 Screening for a reliable source of YAT and purification

**Fig. 5:**
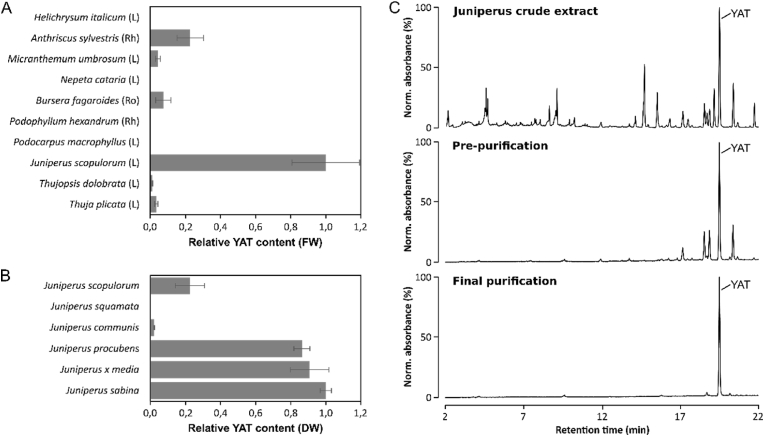
**YAT sourcing from plant material.** (A) Screening of YAT content in different plants (L: leaves; Ro: roots; Rh: rhizomes). Methanolic extraction of lignans was directly performed on fresh plant material followed by quantification using UPLC-MS/DAD. (B) Comparison of YAT content in needles of selected Juniperus species. Samples were collected and flash-frozen prior to lyophilization. Lignans were then extracted with methanol from dry material before analysis by UPLC-MS/DAD. (C) UV chromatograms at different steps of high-scale YAT purification from *Juniperus* biomass, including crude extracts, pre-purification by silica adsorption and normal-phase chromatography, and final purification by combined normal- and reverse-phase chromatography.

To anticipate scale-up production in bioreactors and the associated demand for large amounts of YAT, we investigated potential plant-based sourcing of this compound. Species previously reported to synthesize lignans were screened and compared for their capacity to accumulate YAT (Antúnez Mojica et al., 2016; Sackett, 1993; Renouard et al., 2011). First, no arylnaphthalene lactone lignans were detected in *Helichrysum* or *Nepeta* species (Fig. 5A) and YAT was detected only at trace levels in *Podophyllum* and *Podocarpus*, close to the analytical detection limit.

In contrast, substantial YAT accumulation was measured in the rhizomes of *Anthriscus*, the roots of *Bursera*, the leaves of *Microthemum*, and the needles of several gymnosperms, including *Thuja*, *Thujopsis*, and *Juniperus*. Among these taxa, *Juniperus* exhibited the highest YAT levels and emerged as the most promising biological source. This genus is widely distributed, highly resilient, and its ability to accumulate YAT in aerial tissues enables repeated harvesting without plant destruction, while providing large amounts of biomass. Based on these observations, YAT accumulation was further assessed across multiple *Juniperus* species to account for genetic variability (Fig. 5B). While some species, such as *J. squamata* and *J. communis*, accumulated little to no detectable YAT, *J. × media*, *J. procumbens*, and *J. sabina* were identified as the most productive species among those tested.

To evaluate the feasibility of large-scale YAT isolation, a total of 12.8 kg of fresh aerial parts, consisting of a mixture of different *Juniperus* species, was processed. The purification workflow involved solvent extraction to obtain a crude plant extract, followed by a pre-purification step based on impregnation onto silica and subsequent purification achieved through a combination of normal- and reverse-phase chromatography (Fig. 5C). This purification strategy enabled efficient enrichment of YAT from the crude plant extract, resulting in a progressive increase in compound purity throughout the successive chromatographic steps. Ultimately, 12.5 g of YAT were isolated as a pale green amorphous solid with a purity exceeding 80%. Overall, the quantity and quality of the purified compound were sufficient to support subsequent bioreactor experiments, thereby demonstrating the suitability of *Juniperus*-derived biomass as a scalable source of this lignan precursor.

### 3.5 Scale up of 4’dEPT production in bioreactor

In this study, the scale-up experiments were designed as a proof of concept for production at larger volumes in industrial-like cultivation systems, enabling improved control of culture conditions and optimization of process parameters to intensify precursor conversion into the target product. Although strain Eto 3.4 displayed the highest overall productivity at small scale, strain Eto 3.2 was selected for scale-up experiments to disentangle the respective contributions of process engineering and strain genetic background. This choice also enabled a more detailed analysis of the dynamics of metabolic intermediate accumulation and reconsumption under different feeding strategies. A similar approach has previously been successfully applied to the production of the alkaloid vindoline, highlighting the strong impact of culture operation optimization on bioconversion yield and productivity (Kulagina et al., 2021).

To validate the process scalability, strain Eto 3.2 was therefore cultivated under different operational conditions/modes, while maintaining a common two-phase cultivation pattern consisting of a pure growth phase followed by a dedicated bioconversion phase. Three configurations were thus evaluated. First, 250 mL baffled Erlenmeyer flask cultures were operated with a single precursor pulse (20 mg.L^−1^) to initiate bioconversion, under uncontrolled pH and oxygen conditions, providing a readily transferable screening format. Second, a 2 L stirred tank bioreactor culture was conducted with three successive precursor pulses (20 mg.L^−1^ each) applied at regular intervals to intensify production. This approach was designed to confirm previous observations showing that intermittent feeding at low precursor concentrations can improve both yield and productivity in heterologous pathways sensitive to cofactor availability and metabolic stress, while limiting precursor accumulation and the associated solvent dilution effect (Kulagina et al., 2021). Third, a continuous and constant YAT feeding was implemented in the bioreactor, supplying an overall amount equivalent to the three pulses (60 mg.L^−1^) over the same time window than pulses, with the aim of maintaining a low YAT concentration, which generally favour more complete bioconversion and reduce intermediate accumulation and by-product accumulation (Kulagina et al., 2021).

Bioreactor cultivations were conducted in fed-batch mode under tightly controlled pH, temperature and oxygen conditions, based on previously optimized strategies for precursor bioconversion into alkaloids using the same parental yeast strain. During the growth phase, identical parameters were applied across all experiments to ensure comparable biomass levels at the onset of production including initial medium composition, feed composition, dissolved pO_2_ and pH set points, and a biomass production strategy maintaining a target growth rate through controlled carbon substrate feed rate. This initial phase was followed by the bioconversion phase during which purified YAT was supplied and converted into 4’dEPT.

To prevent metabolic stress associated with nutrient depletion and to maintain an active primary metabolism during production, additional nutrients were continuously supplied via a concentrated solution containing yeast extract, peptone and glucose. This feeding regime ensured a steady supply of nitrogen and carbon sources, thereby supporting enzyme activity in the heterologous pathway. Such a two-phase cultivation process combined with precursor feeding profiles have been validated for several families of molecules. Under these conditions, maintaining an active central metabolism through continuous glucose feeding below the Crabtree effect threshold, together with controlled precursor supply, maximises target-product productivity and bioconversion yield while limiting the formation of undesirable derivatives (Kulagina et al., 2021).

**Fig. 6:**
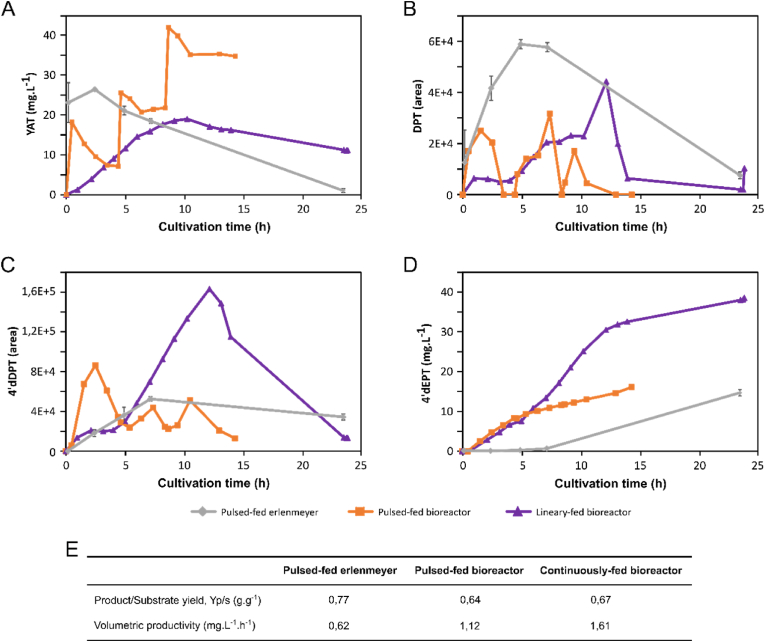
**Scale-up of 4′dEPT production in Eto 3.2 under three operating modes.** Time-course monitoring of extracellular YAT (A), DPT (B), 4’dDPT (C), and 4’dEPT content of Eto 3.2 cultures in pulsed-fed Erlenmeyer baffled flasks (grey diamond), pulsed-fed bioreactor (orange square) and continuously-fed bioreactor (purple triangle). (E) Product (4’dEPT) mass yield on substrate (YAT) and overall volumetric productivity calculated under each condition. Cultures in bioreactors were performed in 2L vessels at 30 °C, pH 6, DO > 20%. Cultures in flasks were performed at 30 °C and initiated at pH 6. All of the cultures were performed on YPD rich media. YAT was fed by one initial pulse in flasks, and by three pulses each 4 h or an equivalent amount through a 12h steady flow in bioreactors.

In pulsed-feed baffled Erlenmeyer flask cultures, the precursor-to-product (Y_p_/_s_) yield reached 0.77 g g^−1^, corresponding to the highest mass conversion efficiency among the tested conditions, although overall volumetric productivity remained limited at 0.62 mg.L^−1^.h^−1^ (Fig. 6E). This reduced productivity likely reflects slower production kinetics, likely resulting from drift in uncontrolled culture parameters. Under these conditions, CYP71BE54 activity appeared particularly affected, leading to DPT accumulation, which in turn strongly limited 4’dEPT synthesis (Fig. 6A–D). When the process was transferred to a pulsed-feed bioreactor, the yield dropped by 17% to 0.64 g g^−1^, suggesting a decrease in substrate conversion efficiency potentially associated with physiological stress induced by the bioreactor or YAT accumulation. Nevertheless, higher precursor availability and tighter biochemical and physico-chemical control of the culture environment resulted in a 81% increase in volumetric productivity, reaching 1.12 mg.L^−1^.h^−1^ (Fig. 6E), consistent with previous observations for vindoline production (Kulagina et al., 2021). Interestingly, sudden YAT accumulation following the second pulse onwards appeared to disrupt 4’dEPT synthesis kinetics, revealing a sensitivity of the pathway to abrupt variation in YAT availability (Fig. 6A–D). In contrast to baffled Erlenmeyer flask cultures, DPT did not accumulate under these conditions despite higher metabolic fluxes, suggesting improved CYP71BE54 activity.

Finally, the constant YAT feeding strategy in the bioreactor did not further improve yield, which remained relatively stable at 0.67 g g^−1^, but markedly enhanced process performance in terms of rate, leading to the highest volumetric productivity observed (1.61 mg.L^−1^.h^−1^; +160%). This strategy effectively mitigated abrupt changes associated with YAT pulses and enabled sustained bioconversion capacity over extended periods. Notably, production kinetics under continuous feeding displayed two distinct phases (Fig. 6A–D). An initial phase corresponding to the YAT feeding period was characterized by a high productivity of 2.42 mg.L^−1^.h^−1^ and increased accumulation of 4’dDPT, reflecting a rapid pathway turnover transiently limited by CYP82D61 activity. This phase was followed by a slower production phase (0.63 mg.L^−1^.h^−1^ on average) despite the presence of residual YAT in the medium. During this second phase, the cells retained a strong capacity to efficiently and preferentially reconsume accumulated intermediates to sustain 4’dEPT synthesis, a phenomenon also observed in the other cultures.

Overall, exploring these different cultivation strategies for YAT bioconversion into 4’dEPT highlights the critical importance of controlling both culture operation and cellular environment. Tight biochemical and physico-chemical control of the culture broth thus significantly improved process productivity by limiting DPT accumulation, even though overall yield decreased. Continuous precursor feeding further intensified bioconversion kinetics, particularly during the feeding phase, notably by limiting YAT accumulation and revealing a strong cell capacity to reconsume 4’dDPT toward 4’dEPT synthesis. This increase in productivity for a lignan precursor is consistent with previous observations obtained for the bioconversion of tabersonine into vindoline (Kulagina et al., 2021). This thus supports the transferability of this scale-up strategy to other plant specialized metabolites produced via heterologous pathways in yeast. However, these results also reveal a trade-off between limited yield and kinetic intensification of the process, as yield stabilization appears to rely on continuous YAT feeding.

### 3.6 Optimization of culture conditions and feeding strategies to enhance YAT bioconversion

**Fig. 7:**
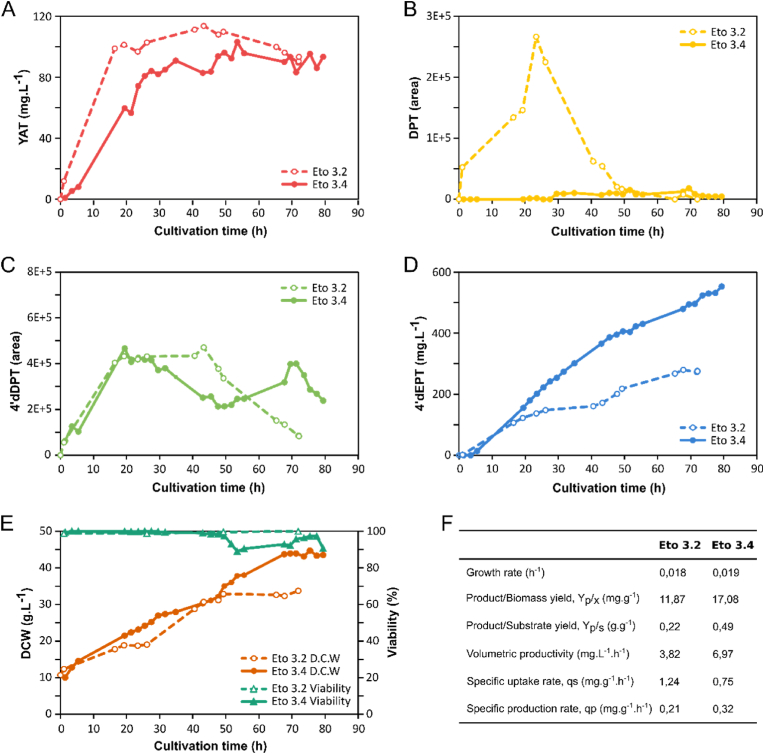
**Optimization of Eto 3.2 and Eto 3.4 culture conditions in bioreactor to maximize YAT bioconversion in 4’dEPT.** Time-course monitoring of extracellular YAT (A), DPT (B), 4’dDPT (C), and 4’dEPT (D) content, and of dried cell weight (DCW, brown circle) and viability (green triangle) (E) of Eto 3.2 (dashed line with open circles) and Eto 3.4 (solid line with filled circles) cultures. (E) Monitoring of dried cell weight (DCW, brown circle) and viability (green triangle). (F) Comparison of the growth rate, the product (4’dEPT) mass yield on biomass (D.C.W), the product (4’dEPT) mass yield on substrate (YAT), the overall volumetric productivity, the specific YAT uptake rate and specific 4’dEPT production rate for both strain. Cultures were performed on YPD in 2L bioreactors at 30 °C, pH 6, DO > 20%. Fresh concentrate YPD and a total of 2 g of YAT were fed continuously for both experiments.

In this section, strain Eto 3.4 that displayed the highest bioconversion capacity at small scale, was investigated under optimized culture conditions to maximize 4’dEPT production, while strain Eto 3.2 was used as a reference to confirm, under intensive process conditions, the impact of the previously identified genetic optimization. Both strains were cultivated in 2 L stirred tank bioreactors operated in fed-batch mode under controlled physicochemical conditions, applying the best production strategy established during the scale-up study. Cultivations consisted of a growth phase followed by a bioconversion phase during which fresh nutrient medium and a total of 2 g of YAT were continuously supplied. This feeding strategy was designed to maintain precursor concentrations below 100 mg.L^−1^ and to prevent abrupt fluctuations in YAT availability (Fig. 7A).

During the bioconversion phase, both strains exhibited comparable growth over the first two days, corresponding to a specific growth rate of 0.018 h^−1^ for strain Eto 3.2 and 0.019 h^−1^ for strain Eto 3.4. Beyond this point, growth of strain 3.2 appeared progressively impaired, in contrast to strain Eto 3.4 (Fig. 7E). Interestingly, the viability of strain Eto 3.4 transiently decreased below 90% after 50 h before subsequently recovering, suggesting that this strain displays sufficient robustness to withstand the stressful conditions associated with intensive production conditions. Strain Eto 3.2 excreted DPT, which accumulated up to a maximum at 23 h before being fully reconsumed by 50 h, in agreement with the observations from the scale-up experiments (Fig. 6B; Fig. 7B). In contrast, no detectable DPT accumulation was observed in strain 3.4, likely owing to the presence of two additional copies of CYP71BE54, which redirected metabolic flux downstream toward 4’dEPT, without causing further 4’dDPT accumulation (Fig. 7C). As a consequence, 4’dEPT production was markedly enhanced in strain Eto 3.4, reaching 553 mg.L^−1^ at the end of the culture compared with 273 mg.L^−1^ for the reference strain (Fig. 7D). This improvement in titer translated into an approximately two-fold increase in bioconversion yield reaching 0.49 g g^−1^ in strain Eto 3.4 versus 0.22 g g^−1^ for the Eto 3.2 reference strain (Fig. 7F). These results highlight the challenge of maintaining high bioconversion yields under intensive culture conditions, as already observed during the scale-up study (Fig. 6E).

From a kinetic standpoint, the improved yield observed in strain Eto 3.4 can be attributed to a 52% increase in the specific 4’dEPT production rate relative to strain Eto 3.2, reaching 0.32 mg g^−1^.h^−1^. This effect was accompanied by an unexpected 40% decrease in the specific YAT consumption rate, which dropped to 0.75 mg g^−1^.h^−1^ (Fig. 7F). The enhanced specific production rate observed in strain Eto 3.4 is consistent with the higher genomic copy number of CYP71BE54, supporting an improvement of the metabolic flux toward 4’dEPT. Similar benefits of balancing endogenous and heterologous pathway capacities have previously been reported for geranylgeraniol production (Song et al., 2017) and alkaloid biosynthesis (Kulagina et al., 2021). Altogether, these findings demonstrate the importance of optimizing the copy number of 2ODD and CYP71BE54 to achieve improved biosynthetic flux distribution within the heterologous pathway. Finally, the most pronounced improvement was observed at the level of process productivity, which reached 6.97 mg.L^−1^.h^−1^ in strain Eto 3.4, corresponding to a 83% increase compared with strain Eto 3.2 (3.82 mg.L^−1^.h^−1^). Notably, process optimization relative to the scale-up experiment had already led to a 137% productivity increase for strain Eto 3.2.

Overall, these optimization results on the production system are consistent with the observations from the scale-up study, highlighting a strong potential for enhancing the bioconversion rate of YAT into 4’dEPT, while also illustrating the challenge of maintaining maximal bioconversion yield under intensified production conditions. More broadly, this work emphasizes the importance of coupling genetic optimization of the producing strain with optimization of the process engineering strategies for the efficient bioproduction of plant-derived specialized metabolites in yeasts.

### 3.7 Chemical glycosylation step and formal synthesis of etoposide

**Fig. 8:**
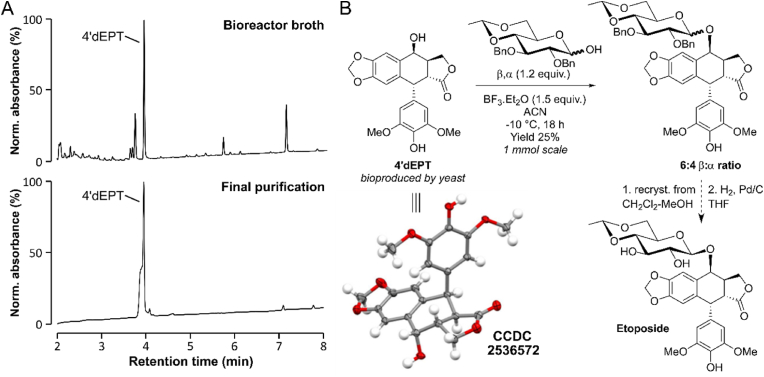
**Purification and chemical glycosylation of bio-produced 4′dEPT for etoposide synthesis.** (A) UV chromatograms of the broth from 1.4L-bioreactor culture of YAT-fed Eto 3.4 strain, used for 4′dEPT purification and purified product obtained by liquid/liquid extraction with dichloromethane followed by reverse-phase chromatography. (B) Schematic of 4′dEPT chemical glycosylation and subsequent formation of etoposide. The structure of 4′dEPT was confirmed by ^1^H NMR, ^13^C NMR (Supplemental Figs. S1 and S2), and crystallography (displacement ellipsoids are drawn at the 50% probability level; deposition number 2536572 contains the supplementary crystallographic data, Supplemental Fig. S5).

With the objective of purifying 4′dEPT from fermentation broths for subsequent chemical glycosylation, two 1.4 L reactor runs were processed, corresponding to an estimated cumulative production of 920 mg of 4′dEPT. Downstream processing, combining liquid–liquid extraction and reverse-phase C18 purification, enabled efficient enrichment of the target compound and resulted in the isolation of 885 mg of purified 4′dEPT (Fig. 8A). The identity and structural integrity of the isolated compound were confirmed by spectroscopic analyses, including ^1^H-NMR (Supplemental Fig. S1), ^13^C-NMR (Supplemental Fig. S2), and by X Ray analysis (Fig. 8B).

To demonstrate the feasibility of producing etoposide from bioproduced 4′dEPT, the latter was subjected to a BF_3_·OEt_2_-mediated coupling with 4,6-*O*-ethylidene-2,3-*O*-dibenzyl-D-glucose. The reaction was conducted on a 1.0 mmol scale. The key step of this sequence is the glycosylation, which proceeds via a stereoselective nucleophilic attack of the aglycone benzylic hydroxyl group on an anomeric oxocarbenium ion intermediate. An anomeric ratio of 6:4, favoring the *β*-isomer, was obtained (Fig. 8B, Supplemental Figs. S3 and S4). Subsequent formal recrystallization, followed by protecting-group removal under Dillon’s conditions (Silverberg et al., 2000) may afford the anticancer agent etoposide. Altogether, these results demonstrate that 4′dEPT produced in engineered yeast bioreactors can be efficiently isolated and chemically converted into an important drug, thereby validating the feasibility of an integrated biotechnological route toward sustainable etoposide production.

## 4 Conclusion

In this work, we established an integrated biotechnological strategy enabling the sustainable production of advanced biosynthetic intermediates of the anticancer drug etoposide in yeast cell factories. By combining pathway engineering, optimization of gene copy number and implementation of compatible CPRs, we successfully reconstructed an efficient heterologous route for the conversion of YAT into 4′dEPT in yeast. Progressive strain optimization enabled substantial improvements in metabolic flux distribution, resulting in enhanced titers and accelerated production kinetics.

Beyond strain engineering, this study highlights the critical contribution of process engineering to overall system performance. Systematic investigation of precursor feeding strategies and cultivation conditions in controlled bioreactor environments demonstrated that tight regulation of culture parameters and dynamic control of substrate availability are key determinants of productivity in this approach. Continuous precursor supply and optimization of early pathway steps notably enabled significant intensification of volumetric productivity, while revealing intrinsic trade-offs between conversion yield and process kinetics under intensified conditions.

Importantly, we further demonstrated the feasibility of coupling bioproduction in cell factories with downstream semisynthetic conversion by successfully isolating bioreactor-derived 4′dEPT and transforming it into etoposide. In parallel, the identification of resilient plant resources capable of accumulating high levels of YAT provides a complementary strategy for securing precursor supply at scale.

In conclusion, these results validate the concept of a hybrid production platform integrating controlled plant biomass sourcing, engineered yeast cell factories and chemical transformation steps. Such a global strategy offers promising biotechnological perspectives for establishing robust and flexible supply chains for high-value plant-derived pharmaceuticals. In the longer term, further optimization of metabolic pathway efficiency, precursor logistics and fermentation processes may enable fully controlled and scalable production systems, contributing to improved access to essential anticancer therapeutics while reducing pressure on natural plant resources.

## CRediT authorship contribution statement

**Nicolas Gautron:** Writing – original draft, Methodology, Investigation, Formal analysis, Data curation. **Jennifer Perrin:** Methodology, Investigation, Formal analysis, Data curation. **Ana Luisa Lopez-Vazquez:** Methodology, Investigation, Formal analysis, Data curation. **Céline Melin:** Methodology, Investigation, Formal analysis, Data curation. **Marianne Unlubayir:** Methodology, Investigation, Formal analysis, Data curation. **Killian Tiger:** Methodology, Investigation, Formal analysis. **Cyril Nicolas:** Methodology, Investigation, Formal analysis. **Clément Cuello:** Investigation, Formal analysis. **Marc Clastre:** Investigation, Conceptualization. **Nicolas Papon:** Investigation, Conceptualization. **Nathalie Giglioli-Guivarc’h:** Investigation, Funding acquisition, Conceptualization. **Loïc Guillonneau:** Investigation, Conceptualization. **Isabelle Gillaizeau:** Writing – original draft, Methodology, Investigation, Funding acquisition, Formal analysis, Conceptualization. **Christophe Hano:** Writing – original draft, Methodology, Investigation, Funding acquisition, Formal analysis, Conceptualization. **Marc Jillian:** Writing – review & editing, Writing – original draft, Supervision, Methodology, Investigation, Funding acquisition, Formal analysis, Conceptualization. **Sébastien Besseau:** Writing – review & editing, Writing – original draft, Supervision, Methodology, Investigation, Funding acquisition, Formal analysis, Conceptualization. **Vincent Courdavault:** Writing – review & editing, Writing – original draft, Supervision, Project administration, Methodology, Investigation, Funding acquisition, Formal analysis, Conceptualization.

## Funding

This work was supported by the 10.13039/501100001665Agence Nationale de la Recherche (ANR; project MIADIM – ANR-25-CE43-6607), la Ligue contre le cancer (Cell4Life and Yeast4life projects) and the Région Centre-Val de Loire (10.13039/501100012495ARD CVL Biopharmaceutical program of the Région Centre-Val de Loire [ETOPOCentre project]; and APR-IR - ScaleBio project).

## Declaration of competing interest

The authors declare the following financial interests/personal relationships which may be considered as potential competing interests: LG is Head of R&D at Axyntis. The authors declare that they have no known competing financial interests or personal relationships that could have appeared to influence the work reported in this paper.

## Data Availability

Data will be made available on request.
