# Supplementary material for: A yeast-based platform for etoposide production via yatein bioconversion
Source: Metab Eng Commun. 2026 May 22;22:e00280. doi: 10.1016/j.mec.2026.e00280 (PMC13234597; doi:10.1016/j.mec.2026.e00280)
Supplement: Multimedia component 1 [file mmc1.docx]

**Supporting information**

**A yeast cell factory for etoposide production**

Nicolas Gautron^1*^, Jennifer Perrin^1*^, Ana Luisa Lopez-Vazquez^1^, Céline Melin^1^, Marianne Unlubayir^1^, Killian Tiger^2^, Cyril Nicolas^2^, Clément Cuello^3^, Marc Clastre^1^, Nicolas Papon^4^, Nathalie Giglioli-Guivarc’h^1^, Loïc Guillonneau^5^, Isabelle Gillaizeau^2^, Christophe Hano^2^, Marc Jillian^1#^, Sébastien Besseau^1#^, Vincent Courdavault^1#^

**Supplemental Table S1:** Primers used in this study

| **Name** | **Sequence 5’->3’** |
| --- | --- |
| ATR2_fw | CTGAGAACTAGTTCGGTCATGAAAAACATGATGAATTATAAATTAAAAC |
| ATR2_rev | CTGAGAACTAGTTTACCATACATCTCTAAGATATCTTCC |
| Ph2ODD_fw | CTGAGAACTAGTTCGGTCATGGGTTCTACAGCACCCCT |
| Ph2ODD_rev | CTGAGAACTAGTTCATGCACCTGTGTACGCCT |
| PhCYP71BE54_fw | CTGAGAACTAGTATGGAGTTCCTTTCATTTCCC |
| PhCYP71BE54_rev | CTGAGAACTAGTCTATTCTTTTGTAGTCGATCTTTCC |
| PhCYP82D61_fw | CTGAGATCTAGAATGGATTCCCTGCACTGCCT |
| PhCYP82D61_rev | CTGAGATCTAGACTAGACAAAACATTTTGGAGAGAT |
| CrCPR2_fw | CTGAGAGCTAGCTCGGTCATGGATTCTAGCTCGGAGAAGTTGTC |
| CrCPR2_rev | CTGAGAGCTAGCTCACCAGACATCTCGGAGATACC |
| PhCPR2_fw | CTGAGAACTAGTTCGGTCATGCAATCAAGCTCAGCTTTC |
| PhCPR2_rev | CTGAGAACTAGTTCACCATACGTCACGGAGATATC |
| CYP82D61opt_fw | CTGAGAACTAGTTCGGTCATGGATTCATTACACTGTTTGGAAACTC |
| CYP82D61opt_rev | CTGAGAACTAGTTTAAACAAAGCACTTTGGGGATATTC |
| CYP82D61opt_bis_fw | CTGAGAACTAGTTCGGTCATGGATTCGTTACATTGCTTAGAAACG |
| CYP82D61opt_bis_rev | CTGAGAACTAGTTTACACAAAGCACTTCGGTGAAATC |

**Supplemental Table S2:** Plasmid constructions used as templates for CRISPR-Cas9 integration of expression cassettes in yeast genome hotspots

| **Plasmid name** | **Expression cassettes** | **Integration locus** |
| --- | --- | --- |
| pDPTA125-ATR2 | pTEF1-ATR2-tADH1 | XII-5 |
| pDPTA125-CrCPR2 | pTEF1-CrCPR2-tADH1 | XII-5 |
| pDPTA125-PhCPR2 | pTEF1-PhCPR2-tADH1 | XII-5 |
| pDPAC124-Ph2ODD/PhCYP71BE54 | pACT1-PhCYP71BE54-tCYC1 | XII-4 |
|  | pTDH3-Ph2ODD-tPGI1 |  |
| pPEAC115-Ph2ODD/PhCYP71BE54 | pACT1-Ph2ODD-tCYC1 | XI-5 |
|  | pPGK1-PhCYP71BE54-tENO2 |  |
| pPETA102-Ph2ODD/PhCYP71BE54 | pTEF1-PhCYP71BE54-tADH1 | X-2 |
|  | pPGK1-Ph2ODD-tENO2 |  |
| pDPTA111-Ph2ODD/PhCYP71BE54 | pTEF1-Ph2ODD-tADH1 | XI-1 |
|  | pTDH3-PhCYP71BE54-tPGI1 |  |
| pPETA104-PhCYP82D61 | pTEF1-PhCYP82D61-tADH1 | X-4 |
| pPETA104-PhCYP82D61opt | pTEF1-PhCYP82D61opt-tADH1 | X-4 |
| pPETA104-PhCYP82D61opt_bis | pTEF1-PhCYP82D61opt_bis-tADH1 | X-4 |
| pPETA104-PhCYP82D61opt/PhCYP82D61opt | pTEF1-PhCYP82D61opt-tADH1 | X-4 |
|  | pPGK1-CYP82D61opt-tENO2 |  |
| pPETA104-PhCYP82D61/PhCYP82D61opt | pTEF1-PhCYP82D61-tADH1 | X-4 |
|  | pPGK1-PhCYP82D61op-tENO2 |  |
| pDPAC112-PhCYP82D61opt_bis/PhCYP82D61opt_bis | pACT1-PhCYP82D61opt_bis-tCYC1 | XI-2 |
|  | pTDH3-PhCYP82D61opt_bis-tPGI1 |  |
| pDPAC113-PhCYP82D61/PhCYP82D61opt | pACT1-PhCYP82D61-tCYC1 | XI-3 |
|  | pTDH3-PhCYP82D61opt-tPGI1 |  |

**Supplemental Table S3:** Yeast strains

| **Strain** | **Expression cassettes and locus of integration used** |
| --- | --- |
| Eto 1.0 | XII-4::pACT1-PhCYP71BE54-tCYC1 - pTDH3-Ph2ODD-tPGI1 |
|  | X-4::pTEF1-PhCYP82D61-tADH1 |
| Eto 1.1 | XII-5::pTEF1-ATR2-tADH1 |
|  | XII-4::pACT1-PhCYP71BE54-tCYC1 - pTDH3-Ph2ODD-tPGI1 |
|  | X-4::pTEF1-PhCYP82D61-tADH1 |
| Eto 1.2 | XII-5::pTEF1-CrCPR2-tADH1 |
|  | XII-4::pACT1-PhCYP71BE54-tCYC1 - pTDH3-Ph2ODD-tPGI1 |
|  | X-4::pTEF1-PhCYP82D61-tADH1 |
| Eto 1.3 | XII-5::pTEF1-PhCPR2-tADH1 |
|  | XII-4::pACT1-PhCYP71BE54-tCYC1 - pTDH3-Ph2ODD-tPGI1 |
|  | X-4::pTEF1-PhCYP82D61-tADH1 |
| Eto 2.1 | XII-5::pTEF1-PhCPR2-tADH1 |
|  | XII-4::pACT1-PhCYP71BE54-tCYC1 - pTDH3-Ph2ODD-tPGI1 |
|  | X-4::pTEF1-PhCYP82D61opt-tADH1 |
| Eto 2.2 | XII-5::pTEF1-PhCPR2-tADH1 |
|  | XII-4::pACT1-PhCYP71BE54-tCYC1 - pTDH3-Ph2ODD-tPGI1 |
|  | X-4::pTEF1-PhCYP82D61opt_bis-tADH1 |
| Eto 2.3 | XII-5::pTEF1-PhCPR2-tADH1 |
|  | XII-4::pACT1-PhCYP71BE54-tCYC1 - pTDH3-Ph2ODD-tPGI1 |
|  | X-4::pTEF1-PhCYP82D61opt-tADH1 - pPGK1-PhCYP82D61opt-tENO2 |
| Eto 2.4 | XII-5::pTEF1-PhCPR2-tADH1 |
|  | XII-4::pACT1-PhCYP71BE54-tCYC1 - pTDH3-Ph2ODD-tPGI1 |
|  | XI-2::pACT1-PhCYP82D61opt_bis-tCYC1 - pTDH3-PhCYP82D61opt_bis-tPGI1 |
| Eto 3.1 | XII-5::pTEF1-PhCPR2-tADH1 |
|  | XII-4::pACT1-PhCYP71BE54-tCYC1 - pTDH3-Ph2ODD-tPGI1 |
|  | X-4::pTEF1-PhCYP82D61-tADH1 - pPGK1-PhCYP82D61op-tENO2 |
|  | XI-3::pACT1-PhCYP82D61-tCYC1 - pTDH3-PhCYP82D61opt-tPGI1 |
|  | XI-2::pACT1-PhCYP82D61opt_bis-tCYC1 - pTDH3-PhCYP82D61opt_bis-tPGI1 |
| Eto3.2 | XII-5::pTEF1-PhCPR2-tADH1 |
|  | XII-4::pACT1-PhCYP71BE54-tCYC1 - pTDH3-Ph2ODD-tPGI1 |
|  | X-4::pTEF1-PhCYP82D61-tADH1 - pPGK1-PhCYP82D61op-tENO2 |
|  | XI-3::pACT1-PhCYP82D61-tCYC1 - pTDH3-PhCYP82D61opt-tPGI1 |
|  | XI-2::pACT1-PhCYP82D61opt_bis-tCYC1 - pTDH3-PhCYP82D61opt_bis-tPGI1 |
|  | XI-5::pACT1-Ph2ODD-tCYC1 - pPGK1-PhCYP71BE54-tENO2 |
| Eto 3.3 | XII-5::pTEF1-PhCPR2-tADH1 |
|  | XII-4::pACT1-PhCYP71BE54-tCYC1 - pTDH3-Ph2ODD-tPGI1 |
|  | X-4::pTEF1-PhCYP82D61-tADH1 - pPGK1-PhCYP82D61op-tENO2 |
|  | XI-3::pACT1-PhCYP82D61-tCYC1 - pTDH3-PhCYP82D61opt-tPGI1 |
|  | XI-2::pACT1-PhCYP82D61opt_bis-tCYC1 - pTDH3-PhCYP82D61opt_bis-tPGI1 |
|  | X-2::pTEF1-PhCYP71BE54-tADH1 - pPGK1-Ph2ODD-tENO2 |
|  | XI-5::pACT1-Ph2ODD-tCYC1 - pPGK1-PhCYP71BE54-tENO2 |
| Eto 3.4 | XII-5::pTEF1-PhCPR2-tADH1 |
|  | XII-4::pACT1-PhCYP71BE54-tCYC1 - pTDH3-Ph2ODD-tPGI1 |
|  | X-4::pTEF1-PhCYP82D61-tADH1 - pPGK1-PhCYP82D61op-tENO2 |
|  | XI-3::pACT1-PhCYP82D61-tCYC1 - pTDH3-PhCYP82D61opt-tPGI1 |
|  | XI-2::pACT1-PhCYP82D61opt_bis-tCYC1 - pTDH3-PhCYP82D61opt_bis-tPGI1 |
|  | X-2::pTEF1-PhCYP71BE54-tADH1 - pPGK1-Ph2ODD-tENO2 |
|  | XI-1::pTEF1-Ph2ODD-tADH1 - ptDH3-PhCYP71BE54-tPGI1 |
|  | XI-5::pACT1-Ph2ODD-tCYC1 - pPGK1-PhCYP71BE54-tENO2 |


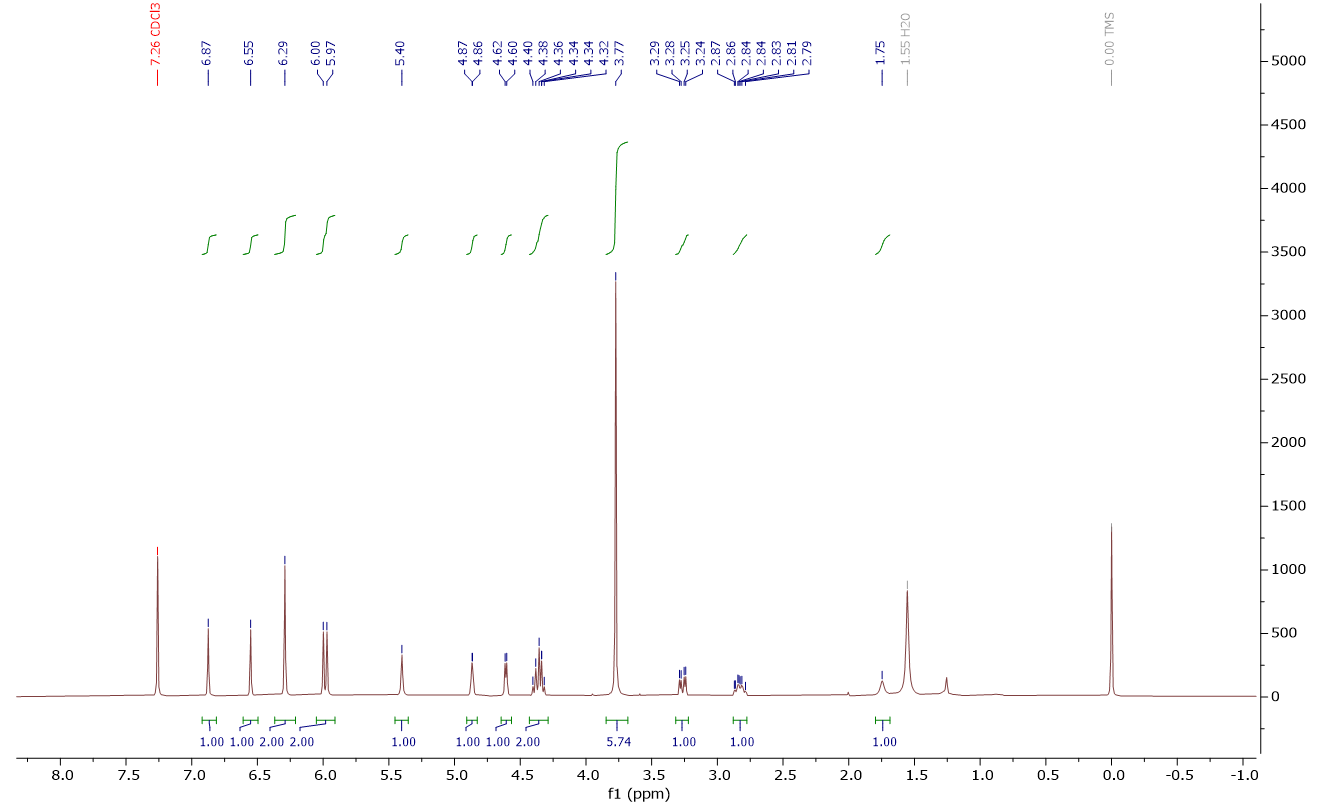


**Supplemental figure S1:** ^1^H NMR spectra (400 MHz, CDCl_3_) of 4’dEPT

 
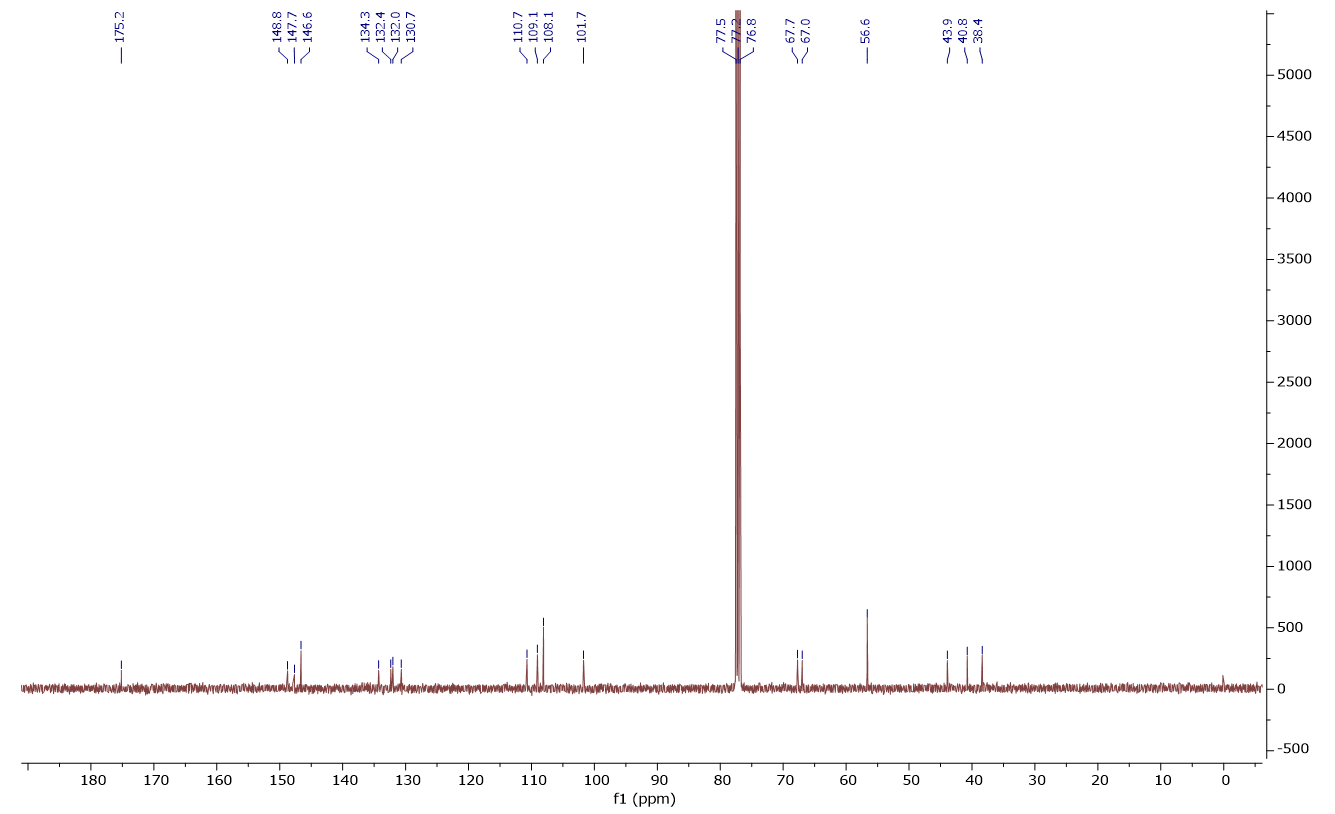


**Supplemental figure S2:** ^13^C NMR spectra (101 MHz, CDCl_3_) of 4’dEPT

  
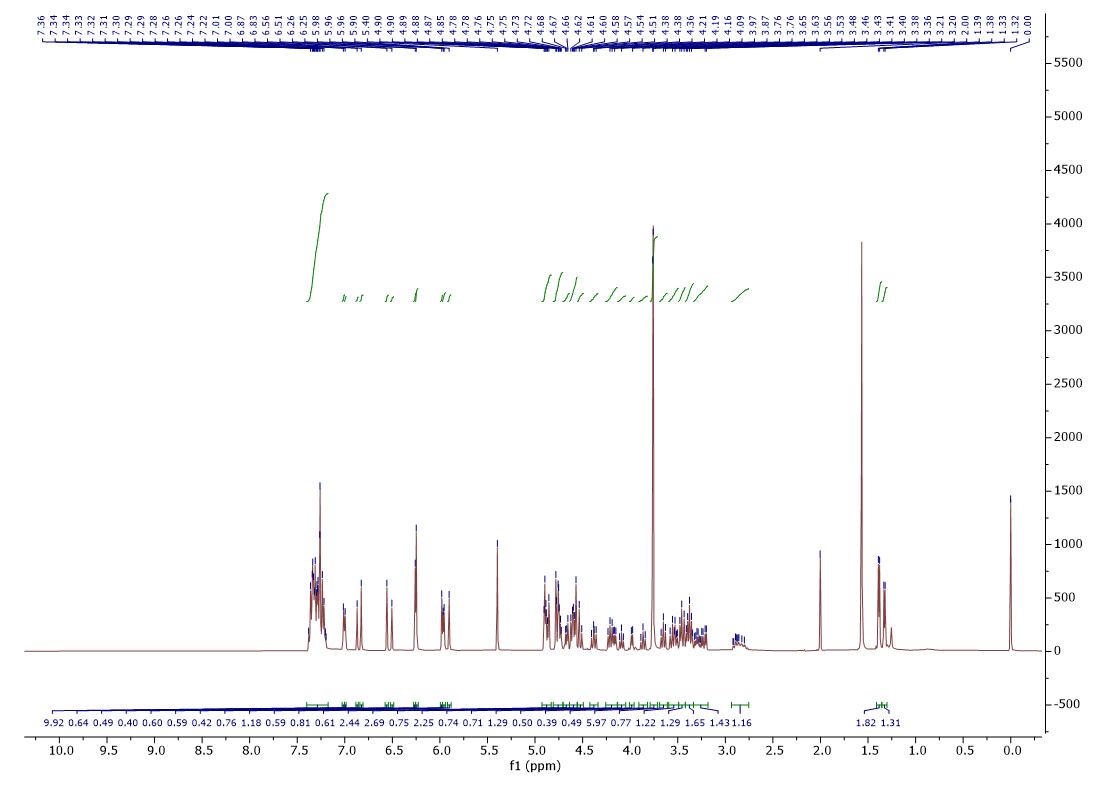


**Supplemental figure S3:** ^1^H NMR spectra (400 MHz, CDCl_3_) of anomeric mixture β/α in a 6:4 ratio


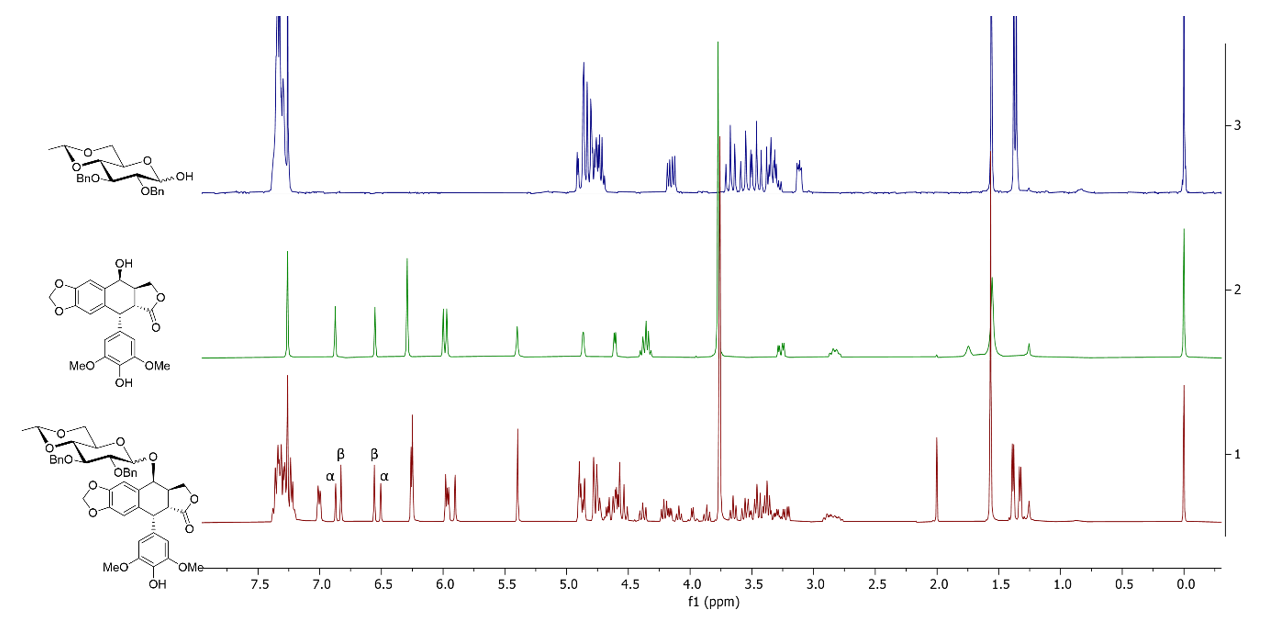


**Supplemental figure S4:** Stacked ^1^H NMR spectra of the starting materials and the glycosylation product.

**Supplemental figure S5:** 4’dEPT crystallographic data


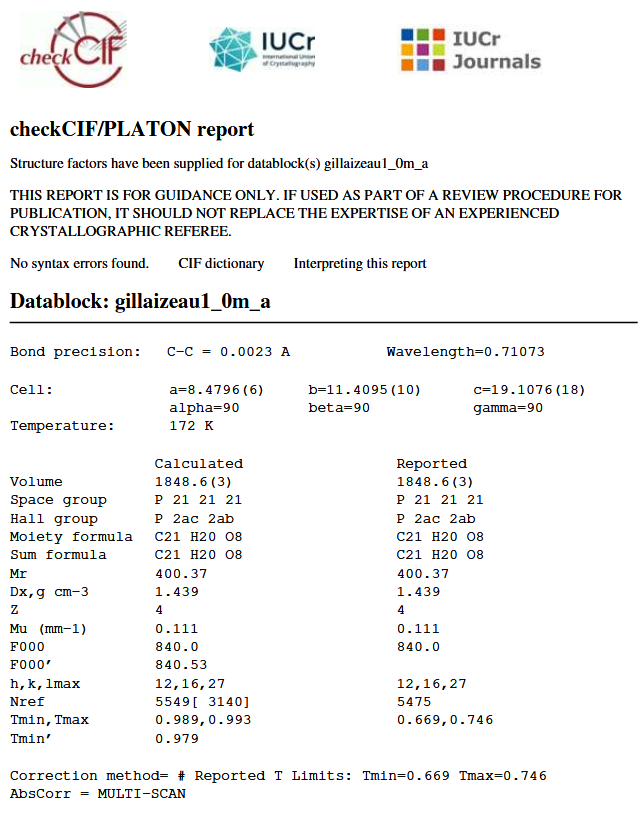


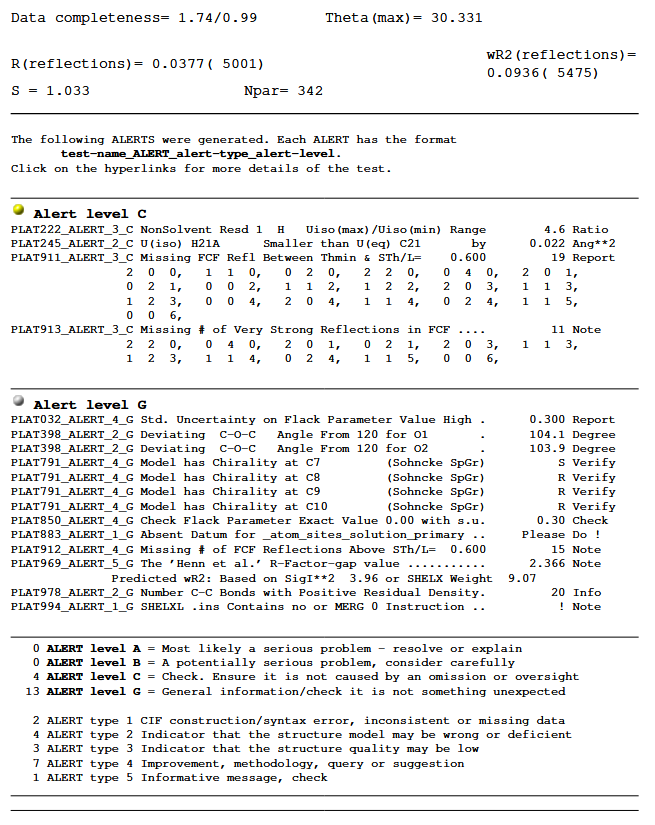


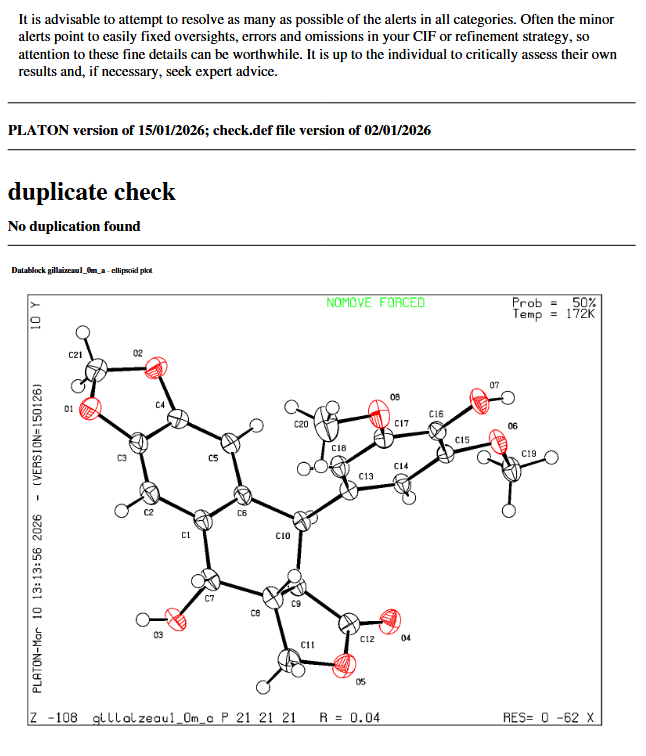


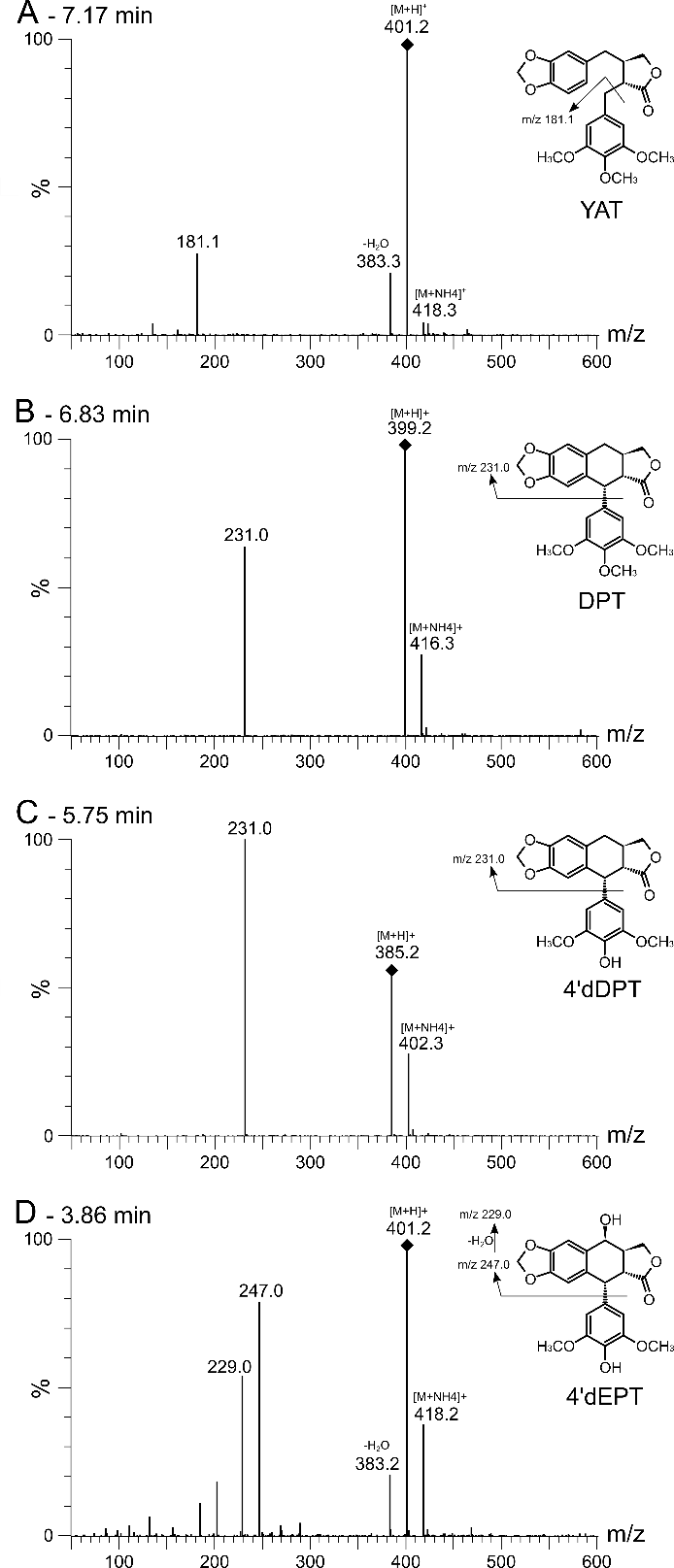


**Supplemental figure S6: MS profile of UV chromatograms peaks at 7.17, 6.83, 5.75 and 3.86 min of retention time in Figure 2.** The expected protonated molecular ion is observed for every compound, but is prone to fragmentation at the C1’/C7’ position, releasing a benzyl ring from the core structure as  observed for arylnaphthalene lactone lignan standards.

>CYP82D61_opt

ATGGATTCATTACACTGTTTGGAAACTCTTTTATTAGGTTTTTTCGTTTTGCTTCCATGTTTTTTTTACTTTGTTTGGAAAAAACCAAATAATAAAATTAAGGAACCGCCCCAACCTGCTGGGGCGTGGCCAATTATAGGACATTTACATTTGCTTGCTAGAGGCGACTTACCTCATAAGATTCTAAGTAGTTTCGCAGATAAAAATGGTCCAGTCTTTAAGATCCAATTGGGTGTTCATCAAGCATTAGTCGTAAATAACTCTGAAATCGCCAAAGAGTGCTTTACTACCAACGATAGATTCTTTTTGAATAGACCGTCAGGTGTTGCTGCAAAAATTATGGGGTATAACTATGTTATGTTGGGCGTGGCACCTTACGGCCCCTATTGGAGGGATATGAGAAAAATAATCATGCTAGAATTTTTGTCTAACAGGCGCTTACAGAGCTTAAAGCATGTTTGGCATTCTGAAATCTCAATCTCTTCCAAAGAACTATACAAACTATGGGAAACACAAAACATTGATTTCTGCCTTGTAGACATGAAGCAATGGTTGGCGGATTTAACGTTGAATATGTCGGTGAAGATGGTTGTTGGGAAAAGGTTCTTCGGTTCAGCGTCCGCTTCTGCCTGTGAGGAAACGGAAAGCAGTAATTGTCCAAAGACACTTCGCAACATGTTCAGACTCATGGGAAGCTTCGTCTTGTCGGACTATTTACCATACCTGCGATGGTTGGACCTCGGTGGTCACGAAAAGGAAATGAAGAGAACAGTTAAAGAGTTGGATATCTTGTTTAAGGGTTGGTTAGATGAACACAAACGAAAAAGACTTTCGGGTGGCAAGGAGGATGACGATCAGGATTTTATGGACGTCATGCTGAGTATACTGGAAGAATCTAAACTGGGAAATGATGTTGACACGATAAATAAGACCGCATGCCTGGCTATTATTTTGGGCGGTGCAGATACCACCTGGGCTACTTTAACTTGGGCCCTCTCACTACTTCTCAACAACCCCAATGCCCTAAAAAAAGCTCAAGACGAGCTTGACCTACACGTAGGAAGAGATAGAAATGTGGATGAATCAGATCTCGTTAAACTTACTTATATAGACGCCATTATTAAAGAAACTTTGAGACTATATCCACCAGGGCCTCTTTTGGGACCTCGTGTCGTGACAGAGGATTGTACAATTGCTGGTTATCATGTACGTGCTGGAACGAGGTTGATAGTAAATGCATGGAAAATACAGCGTGATCCTTTAGTATGGTCTCAACCACATGAGTACCAGCCGGAAAGATTTTTAGAGAGGGACGTGGATATGAAAGGTCAACATTTTGAGCTCATCCCTTTCGGAAGTGGTCGGAGAGCTTGTCCCGCCATTTCCTTGGCCTTGCAAGTATTACCTCTGACTCTGGCACACATCTTACATGGCTTTGAACTACGTACACCAAATCAAAACAAGGTTGATATGACCGAAACACCTGGTATTGTCCACGCAAAGGCTACACCGTTAGAAGTGCTGGTCGCTCCAAGAATATCCCCAAAGTGCTTTGTTTAA

>CYP82D61_opt_bis

ATGGATTCGTTACATTGCTTAGAAACGTTATTGTTAGGATTCTTTGTTTTGTTGCCTTGTTTCTTTTACTTTGTTTGGAAGAAGCCCAATAACAAAATCAAAGAACCTCCACAACCAGCTGGTGCATGGCCAATAATCGGACACTTACATCTTTTAGCCAGAGGGGATTTACCACACAAAATTTTGTCCAGTTTTGCTGACAAGAATGGTCCAGTGTTTAAGATCCAACTTGGTGTTCATCAAGCCTTAGTTGTAAACAACAGCGAAATTGCCAAAGAATGCTTTACTACAAACGATAGATTCTTCCTGAACAGACCTAGCGGAGTTGCTGCCAAAATAATGGGGTACAATTATGTCATGTTAGGTGTAGCCCCATATGGTCCATATTGGCGTGATATGAGGAAAATAATTATGTTGGAGTTCTTGTCTAATAGACGTCTACAGAGTTTGAAACACGTTTGGCATTCTGAGATATCTATTTCATCGAAGGAACTTTATAAGCTTTGGGAAACCCAAAACATTGACTTCTGTTTAGTGGATATGAAACAGTGGTTAGCAGATCTAACCTTGAACATGTCGGTAAAGATGGTTGTTGGGAAAAGATTCTTTGGCTCAGCTTCTGCGTCTGCTTGTGAAGAGACAGAATCATCAAATTGTCCTAAGACTTTGAGAAACATGTTTAGATTAATGGGCAGTTTCGTCTTGTCTGACTATCTACCCTATTTGAGGTGGCTGGATTTGGGTGGACATGAGAAAGAAATGAAAAGAACCGTTAAGGAGTTGGATATTCTATTTAAAGGTTGGTTGGATGAACACAAAAGGAAAAGATTATCAGGTGGCAAGGAGGACGACGATCAAGACTTTATGGATGTTATGCTATCCATCTTAGAGGAAAGCAAACTGGGAAATGATGTAGACACTATTAATAAGACAGCTTGTTTAGCAATCATTCTAGGTGGCGCAGATACGACGTGGGCAACACTTACATGGGCTTTGTCCTTGCTTCTGAATAATCCCAATGCACTAAAGAAGGCTCAAGATGAGCTTGACCTACATGTCGGCAGAGACAGAAATGTCGATGAATCCGATTTGGTCAAATTGACCTACATAGACGCGATAATCAAAGAAACATTACGTTTGTATCCACCTGGTCCGTTATTGGGTCCTAGAGTTGTCACTGAAGATTGCACAATAGCTGGTTACCATGTTAGAGCTGGAACCAGGCTAATTGTTAATGCATGGAAAATTCAAAGGGACCCTTTGGTATGGTCCCAACCACATGAATACCAACCTGAAAGATTTCTTGAAAGAGATGTGGATATGAAAGGACAGCATTTTGAACTGATTCCTTTCGGCTCTGGTAGAAGAGCGTGTCCGGCTATCAGTTTGGCACTACAAGTATTACCCCTTACTTTGGCTCACATACTGCACGGTTTCGAACTGCGTACTCCAAACCAGAATAAGGTGGATATGACTGAGACTCCAGGGATTGTCCATGCCAAAGCCACTCCGTTGGAAGTGTTAGTAGCACCAAGGATTTCACCGAAGTGCTTTGTGTAA

**Supplemental figure S7:** Codon-optimized sequences of CYP82D61


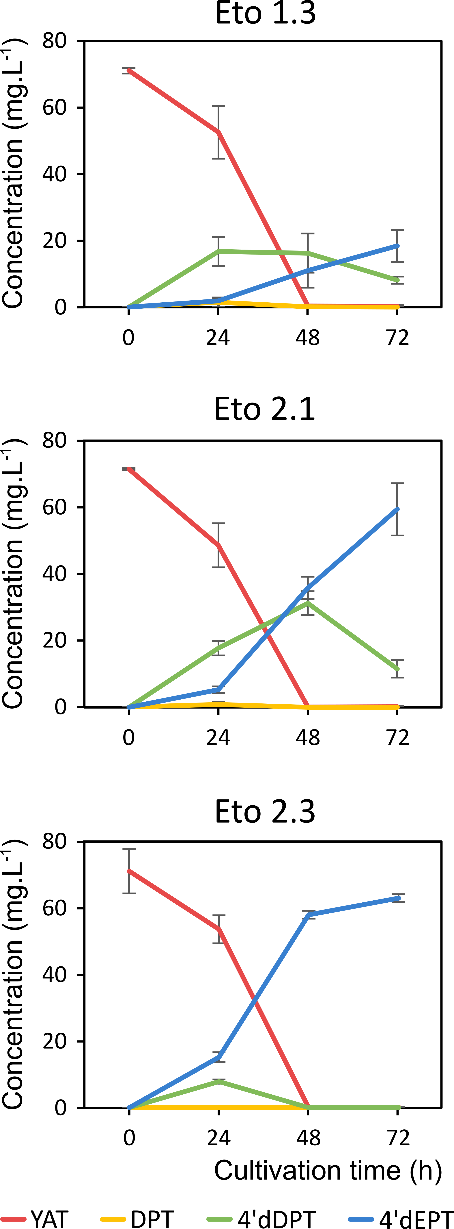


**Supplemental figure S8:** Time-course profiling of lignans in Eto 1.3, 2.1 and 2.3 strains grown during 72 h after addition of 70 mg.L⁻¹ YAT in the culture medium. Error bars represent standard deviation (n = 3 or 4 biological replicates).


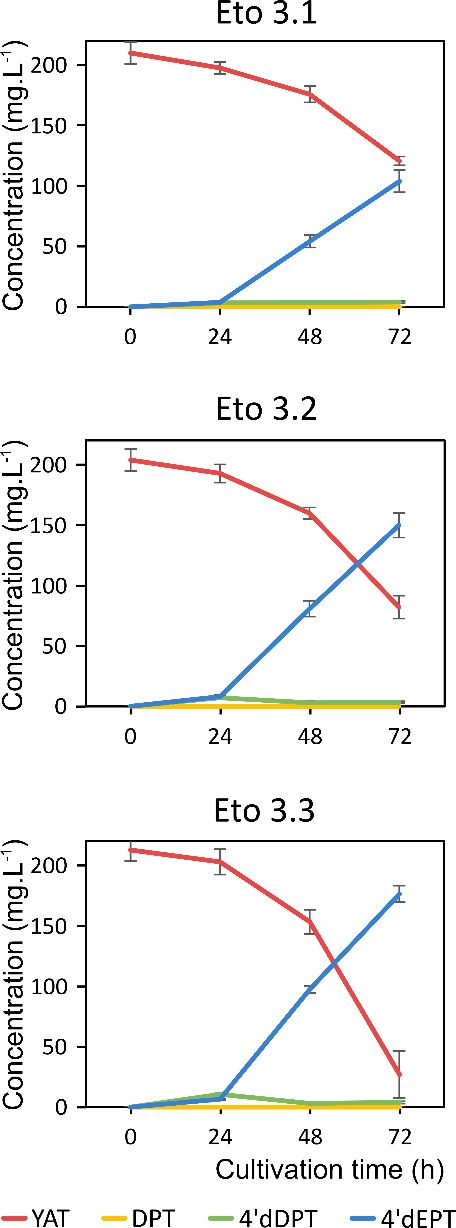


**Supplemental figure S9:** Time-course profiling of lignans in Eto 3.1, 3.2 and 3.3 strains grown during 72 h after addition of 210 mg.L⁻¹ YAT in the culture medium. Error bars represent standard deviation (n = 3 or 4 biological replicates).
